# Radiative Heat Transfer Properties of Fiber–Aerogel Composites for Thermal Insulation

**DOI:** 10.3390/gels11070538

**Published:** 2025-07-11

**Authors:** Mohanapriya Venkataraman, Sebnem Sözcü, Jiří Militký

**Affiliations:** Department of Material Engineering, Faculty of Textile Engineering, Technical University of Liberec, Studentská 1402/2, 46117 Liberec, Czech Republic; jiri.militky@tul.cz

**Keywords:** fibrous aerogels, aerogel composites, heat transfer, radiative heat transfer, FTIR analysis

## Abstract

Fiber–aerogel composites have gained significant attention as high-performance thermal insulation materials due to their unique microstructure, which suppresses conductive, convective, and radiative heat transfer. At room temperature, silica aerogels in particular exhibit ultralow thermal conductivity (<0.02 W/m·K), which is two to three times lower than that of still air (0.026 W/m·K). Their brittle skeleton and high infrared transparency, however, restrict how well they insulate, particularly at high temperatures (>300 °C). Incorporating microscale fibers into the aerogel matrix enhances mechanical strength and reduces radiative heat transfer by increasing scattering and absorption. For instance, it has been demonstrated that adding glass fibers reduces radiative heat transmission by around 40% because of increased infrared scattering. This review explores the fundamental mechanisms governing radiative heat transfer in fiber–aerogel composites, emphasizing absorption, scattering, and extinction coefficients. We discuss recent advancements in fiber-reinforced aerogels, focusing on material selection, structural modifications, and predictive heat transfer models. Recent studies indicate that incorporating fiber volume fractions as low as 10% can reduce the thermal conductivity of composites by up to 30%, without compromising their mechanical integrity. Key analytical and experimental methods for determining radiative properties, including Fourier transform infrared (FTIR) spectroscopy and numerical modeling approaches, are examined. The emissivity and transmittance of fiber–aerogel composites have been successfully measured using FTIR spectroscopy; tests show that fiber reinforcement at high temperatures reduces emissivity by about 15%. We conclude by outlining the present issues and potential avenues for future research to optimize fiber–aerogel composites for high-temperature applications, including energy-efficient buildings (where long-term thermal stability is necessary), electronics thermal management systems, and aerospace (where temperatures may surpass 1000 °C), with a focus on improving the materials’ affordability and scalability for industrial applications.

## 1. Introduction

Aerogels, developed over 60 years ago by Kistler [1], possess remarkable thermal insulating properties due to their unique chemical preparation process, which results in a highly porous material with extremely small pore sizes. These micropores, typically ranging from 5 to 50 nanometers, effectively suppress both convection and gaseous conduction [2,3], making their heat transfer characteristics closely resemble those of evacuated insulation. The macromolecule-like skeletal structure further reduces conduction through the solid phase, contributing to their excellent thermal insulation performance. However, because of their fine structure, aerogels are inherently fragile and are usually transparent to thermal radiation, particularly in the near- to mid-infrared wavelengths [4,5,6]. This transparency significantly undermines their thermal effectiveness at temperatures above ambient, unless radiation-attenuating additives are incorporated. Adding fibers that strongly absorb and scatter infrared radiation greatly enhances the thermal performance of aerogels [7,8,9,10] while simultaneously improving their structural properties. Determining reliable thermophysical data for aerogels, and understanding the physics behind these properties, presents a considerable challenge. Nevertheless, these advancements will ultimately position aerogels as a highly promising material for specialized applications.

Silica aerogels are produced using a sol–gel technique combined with supercritical evaporation technology, resulting in a nanoporous, super-insulating material with exceptional properties. These aerogels are highly promising as thermal insulators due to their remarkably low thermal conductivity, which is less than 0.02 W·m^−1^·K^−1^ under ambient conditions [3,4,11,12,13,14]. However, two major issues limit their application. First, their fragile SiO_2_ solid skeleton is formed from long chains of loosely bonded, amorphous silica nanoparticles, making aerogels brittle. Second, at higher temperatures, the radiative heat transfer increases drastically due to the aerogels’ low extinction coefficients in the 2–8 µm wavelength range [15,16].

To overcome these challenges, various techniques have been explored to strengthen the mechanical properties of aerogels. These methods include the incorporation of polymers, carbon nanotubes, nanowires, and the use of reinforced organic precursors. Despite significant progress, simultaneously optimizing critical properties such as density, surface area, and thermal stability remains a challenge. Recent studies have focused intensively on strengthening silica aerogels using a range of techniques to effectively overcome their inherent limitations. Xue et al. conducted a comprehensive investigation into how different reinforcement strategies—such as the incorporation of polymers, inorganic materials, and fiber-based composites—enhance the mechanical and thermal properties of aerogels [17]. Notably, inorganic reinforcements—such as the incorporation of silica nanowires and ceramic fibers—significantly improve the thermal properties of aerogels and consistently outperform polymer-based reinforcement techniques, as demonstrated by Wang et al. [18]. Furthermore, Zhan et al. focused intensely on aerogels reinforced with silica nanowires, emphasizing their significantly improved mechanical strength and markedly reduced thermal conductivity, making them ideal for applications requiring exceptionally high thermal stability [19]. The potential of fiber-based composites to dramatically enhance the mechanical properties of aerogels has been thoroughly explored, particularly for composites containing glass and ceramic fibers. To further optimize the thermal and mechanical performance of fiber-reinforced silica aerogels, recent studies have carefully fine-tuned fabrication procedures, such as using minimal solvent amounts and precisely controlled temperature aging. Additionally, carbon nanotubes (CNTs) and graphene are widely regarded as the most effective materials for producing ultralight, flexible, and highly conductive aerogels. Their exceptional mechanical strength, ultralow density, excellent elasticity, and superior electrical conductivity, along with their remarkable aspect ratio, make them ideal for a variety of applications. Studies have demonstrated conclusively that carbon-based 1D or 2D nanomaterials in aerogels open up a broad range of promising applications [5,15]. Microscale fibers are commonly integrated into the aerogel matrix to significantly enhance the mechanical strength of silica aerogels [20]. The careful selection of fibers can dramatically reduce infrared radiative transfer by effectively increasing scattering and absorption [21,22]. To significantly enhance the toughness of silica aerogels, composite insulation materials have been successfully synthesized in recent years by combining silica aerogel with conventional insulation materials [23,24,25]. Two notable examples of these reinforcements are xonotlite–aerogel composites and ceramic fiber–aerogel composites. These innovative reinforcement techniques represent a major breakthrough in the development of silica aerogels, expanding their applications to industries such as energy-efficient technology, construction, and aerospace. These advancements greatly enhance the potential for silica aerogels to be more widely adopted in high-performance thermal insulation applications by addressing issues related to mechanical fragility and radiative transparency. To accurately predict and understand the thermal insulation properties of these composites, a heat transfer model is needed that accounts for the structure of the fiber-loaded aerogel. Heat transfer models are generally classified into three major categories. The thermal conductivities of the solid (ks), gas (kg), and radiation (kr) can easily be combined to form the first effective thermal conductivity model, expressed as keff = ks + kg + kr [26,27]. When the pore diameters are smaller than 1 mm, convective heat transfer can generally be considered negligible. The second model, expressed as keff = kc + kr, effectively calculates the effective thermal conductivity (keff) by combining the radiation (kr) and the solid–gas conduction (kc) [26,28,29,30]. To determine the combined solid and gas thermal conductivity, kc, either an empirical model that incorporates parallel and series thermal resistance or a simplified periodic structure based on the real material structure is used [28,31].

The heat transfer mechanisms in silica aerogel and its composites involve solid conduction, gas conduction, and thermal radiation [25,26,27], all of which have been extensively studied in recent years. Due to the micrometer- or nanometer-sized pores in silica aerogel and its composites, natural convection can easily be neglected. On the other hand, thermal radiative heat transfer plays a critical role in these highly effective insulation materials. Studies have strongly indicated that the contribution of radiative heat transfer increases dramatically as the temperature rises [32,33]. Although gas conduction can generally be excluded under vacuum conditions [29], isolating thermal radiation from effective conductivity measurements remains quite challenging. Surprisingly, few studies have truly explored the exact radiative transfer mechanisms in silica aerogels or their composites. Moreover, many previous investigations have largely overlooked the individual processes involved in radiative heat transfer, especially in the context of silica aerogel composite materials. At both ambient and elevated temperatures, radiative absorption and emission ultimately become the dominant heat transfer mechanisms in aerogels. One of the main obstacles for aerogels as thermal insulators is their high transparency to infrared radiation, particularly within the 3–8 µm wavelength range, which allows significant heat to pass through.

In most heat transfer models for monolithic aerogels, conduction and radiation are typically treated as additive contributions, often combined through an effective radiative conductivity. To significantly improve the thermal insulation performance of aerogels, researchers have developed several promising strategies. One effective approach is doping aerogels with carbon, which absorbs infrared radiation and dramatically reduces radiative heat transfer. Another powerful method is incorporating fibers into the aerogel matrix. These fibers not only block the infrared-transparent window, limiting radiation transport, but also reinforce the otherwise brittle structure of monolithic aerogels. The type and concentration of the fibers are critical in terms of optimizing the material’s thermal insulation properties, especially at elevated temperatures. Therefore, a comprehensive understanding of the radiative behavior of fibrous aerogels is crucial for enhancing their effectiveness as thermal insulators [34].

**Figure 1:**
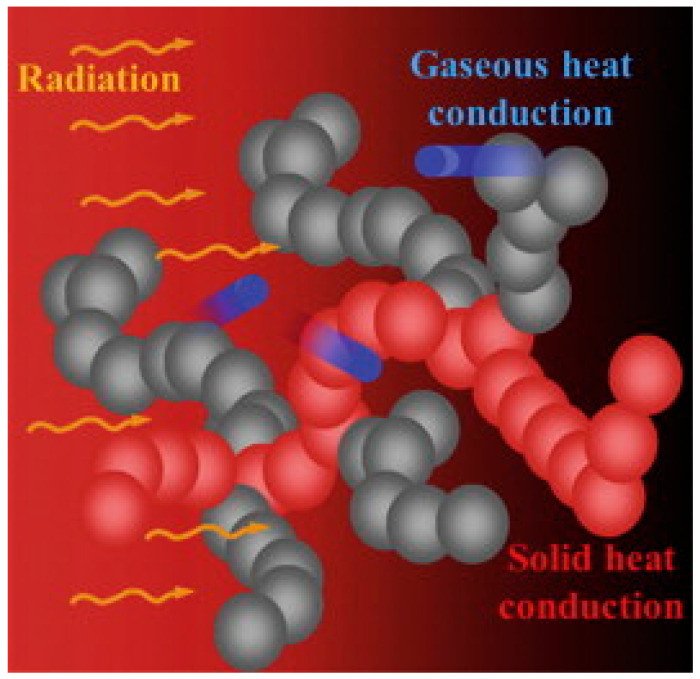
The aerogel material’s linked heat transfer [15].

Optimizing fiber-filled aerogels for different uses needs a clear grasp of the radiative properties of fibers in an aerogel matrix. It also requires understanding radiative heat transfer in the composite material. Models like those by Lee accurately consider the structure of fibrous media. They are useful for examining how fibers interact with the optical properties of aerogels [35,36,37]. These models focus on important factors, including absorption and scattering coefficients, as well as the scattering phase function. These elements are vital for determining the composite’s effective properties. Many studies have looked into calculating radiative heat transfer in fibrous materials. Most use an assumed asymmetry factor to explain the scattering phase function in a two-flux radiative transfer model [38,39]. The development of a new class of aerogel-based composites, referred to as aerogel–fiber composite mats, has been successfully demonstrated by researchers. Padmanabhan et al. [40] employed a simple drying technique to fabricate silica cryogenic glass fiber composites, resulting in high porosity and a significant specific surface area. In comparison to traditional fiber mats, Xue et al. [41] showcased enhanced tensile strength, thermal stability, and insulating properties through their innovative method of fiber mat production. Furthermore, He et al. [42] utilized in situ sol–gel impregnation to produce a fiber mat exhibiting exceptional thermal stability and extraordinarily low thermal conductivity (0.016 W·m^−1^·K^−1^), thereby achieving superior thermal insulation performance. Recent developments in fiber mat technology, which incorporate a higher fiber content, significantly enhance aerogels’ mechanical characteristics while preserving their remarkable thermal insulation capabilities. They differ from traditional aerogel–fiber composites because of these enhancements. Gas molecules will congregate at the interface between neighboring particles because of the nanoscale pore and the nanoscale solid matrix. According to some experts, the so-called solid-like quasi-lattice vibration phenomena might result in an increase in heat transmission [43,44]. In the meantime, as illustrated in Figure 1, the various heat transmission modes inside the aerogel material will combine to create a coupled effect [15].

The goal of this paper is to present a thorough analysis of the radiative heat transfer characteristics of fiber–aerogel composites, emphasizing new advancements, present difficulties, and potential future paths.

## 2. Background and Theoretical Foundations

### 2.1. Radiative Heat Transfer Analysis

Silica aerogels and their composite insulation materials are semi-transparent and isotropic. They can absorb, emit, and scatter thermal radiation. To assess the effects of radiation and conduction in these materials, we must solve the radiative heat transfer equation and the energy conservation equation. However, the radiative heat transfer equation is complex, so its exact form is rarely used in practice. Instead, simplified models are often applied. Common methods include the diffusion approximation with the Rosseland mean extinction coefficient and the two-flux method [32,42,45,46,47,48,49].

Silica aerogel and its composites have enough optical thickness for this study. The insulation layer must be more than 17 mm for silica aerogel and over 9.3 mm for xonotlite-type calcium silicate to meet this requirement. According to the diffusion approximation theory [32,46,50,51,52], when the temperature gradient is small or the optical thickness is large, we can evaluate radiative energy based on local intensity. This intensity is affected by radiation from nearby areas. Under these conditions, we can simplify the integral expression for radiative energy to a diffusion equation similar to Fourier’s law of heat conduction.

(1) $$q_{r}=-k_{r}\frac{\partial T}{\partial x}$$

If the insulation sample is wide enough to be optically thick, you can roughly calculate the radiative thermal conductivity $k_{r}$ using the Rosseland equation [53,54,55,56,57,58]:

(2) $$k_{r}=\frac{16}{3\rho K_{e,m}}\sigma T^{3}$$

where σ = 5.67 × 10^−8^ W/(m^2^ K^4^) is the Stefan–Boltzmann constant, *T* is the medium’s local temperature, ρ is the medium’s bulk density, and $K_{e,m}$ is the Rosseland mean extinction coefficient defined by

(3) $$\frac{1}{K_{e,m}}=\frac{\int_{0}^{\infty }\frac{1}{K_{e,\lambda }}\frac{\partial e_{b,\lambda }}{\partial T}d\lambda }{\int_{0}^{\infty }\frac{\partial e_{b,\lambda }}{\partial T}d\lambda }=\int_{0}^{\infty }\frac{1}{Ke,\lambda }\frac{\partial e_{b,\lambda }}{\partial e_{b}}d\lambda$$

Here, $e_{b,\lambda }$ and $e_{b}$ are the spectral and total emissive power of a blackbody, respectively. Plank introduced the quantum concept for electromagnetic energy to derive $e_{b,\lambda }$

(4) $$e_{b,\lambda }(T,\lambda )=\frac{C_{1}\lambda^{-5}}{e^{C_{2}/\lambda T}-1}$$

where $C_{1}$ = 3.7419 × 10^16^ W m^2^, and $C_{2}$= 1.4388 × 10^−2^ m K. By integrating Equation (4) over all wavelengths, the total radiation $e_{b}$ is obtained as

(5) $$e_{b}(T)=\sigma T^{4}$$

It is important to note that the Rosseland equation is applicable only when the material has sufficient thickness. For a homogeneous material, $K_{e,m}$ represents the intrinsic property of the medium. If $K_{e,\lambda }$ is known, $K_{e,m}$ can be determined using Equation (3), which subsequently allows for the calculation of the radiative heat flux from Equation (2).

### 2.2. Thermal Conductivity and Radiative Heat Transfer

Conduction is the main method of heat transfer in insulating materials, but radiative heat transfer becomes more important at higher temperatures, especially in low-density and porous materials. Improving the performance of insulating materials, especially in high-temperature settings, requires an understanding of the balance between radiative heat transmission and thermal conductivity [59,60].

One key metric for a material’s heat transfer is its thermal conductivity (k). Materials with low thermal conductivity are best for insulation. They stop heat transfer and enhance insulation. A material’s density, microstructure, and chemical makeup all affect its thermal conductivity. When the solid phase, which conducts heat, decreases, higher porosity often leads to lower thermal conductivity. The gas in the pores of insulating materials has much lower thermal conductivity than the solid matrix. Heat transfer mainly occurs through phonon conduction in the solid. Most thermal insulators rely on the interaction between these two phases, which lowers the material’s effective thermal conductivity [60,61].

In materials with micro- or nanostructured shapes, like fibers, foams, and aerogels, radiant heat transmission matters, especially at high temperatures. The Stefan–Boltzmann law explains this process. It states that a surface’s radiative heat flow is proportional to the fourth power of its absolute temperature (T^4^). At high temperatures, materials can emit large amounts of thermal radiation. In materials that are highly porous or low in density, radiative heat transfer can significantly impact overall heat transfer. Here, a material’s emissivity is key. Emissivity measures how well a material emits thermal radiation. Insulating materials with higher emissivity values often radiate more heat, which boosts effective thermal conductivity at elevated temperatures [62,63].

Fiber–aerogel composites have outstanding radiative and thermal conductivity qualities, but they also have some thermal stability traits that are essential for high-temperature applications. When assessing how well fiber–aerogel composites function at high temperatures, thermal stability is a crucial consideration. The material’s resistance to thermal degradation and combustion potential are determined in part by properties like ignition and decomposition temperatures. Applications where materials are subjected to extremely high temperatures require an understanding of these properties.

The thermal stability of certain fiber–aerogel composites has been examined recently:

Polyimide Aerogels: It was discovered that 495 °C was the starting point for the polyimide aerogel matrix material’s breakdown when exposed to nitrogen gas. When compared to the pristine matrix, composites made from this aerogel matrix showed better thermal stability and higher breakdown temperatures [64].
Cellulose–Silica Composite Aerogels: These composites showed remarkable thermal insulation performance, preserving good recovery at 600 °C while providing efficient thermal insulation. Interestingly, they showed excellent thermal stability by not burning in a flame at 1300 °C [65].
Silica Aerogels: Applications needing low thermal conductivity can benefit from silica aerogels because of their high porosity and ultralow thermal conductivity. To improve performance in high-temperature situations, they may need reinforcing because of their poor thermal stability [66].

### 2.3. Fibrous Materials, Aerogels, and Their Composite Structures

Fibers that are several millimeters long and have diameters under 10 μm are often treated as infinitely long circular cylinders. This simplification works well because research shows that when the length-to-diameter ratio (aspect ratio) exceeds 100, the scattering behavior of finite and infinite cylinders becomes similar. As two-dimensional scatterers, the radiative properties of fibrous materials are very sensitive to the fibers’ orientation relative to the medium’s boundaries. Many studies have explored how fiber orientation affects radiative heat transfer in fibrous media. For example, Lee created a radiation model to assess how fiber orientation impacts radiative heat transfer through fibrous materials between two parallel planar diffuse boundaries [67,68]. For randomly oriented fibers, you can find the extinction and scattering coefficients per unit length for a mix of fiber sizes. Average these over all incident directions using the right formulas. These coefficients are key for accurately modeling radiative heat transfer in fibrous media. They consider the complex interactions between incident radiation and the fiber structure, respectively,

(6) $$\mathrm{Extinction}\;\mathrm{Coefficient}:\;K_{f\lambda }=\frac{2f_{vf}}{\pi }\sum_{i=1}^{N}\frac{x_{i}}{r_{fi}}\int_{0}^{\pi /2}Q_{f.e\lambda }(\phi )\cos\phi d\phi$$

(7) $$\mathrm{Scattering}\;\mathrm{Coefficient}:\;\sigma_{f.s\lambda }=\frac{2f_{vf}}{\pi }\sum_{i=1}^{n}\frac{x_{i}}{r_{fi}}\int_{0}^{\pi /2}Q_{f.s\lambda }(\phi )\cos\phi d\phi$$

The scattering phase function for fibrous media accounting for fiber orientation has been formulated by Lee [67]. The scattering cross section of fibers varies with incident angle and the product of the scattering cross section ($\sigma_{f.s\lambda }$) and phase function ${(\rho }_{f\lambda })$ must be considered together. For a medium of randomly oriented fibers, ${<\sigma_{s}P>}_{f\lambda }$, is given by

(8) $$<\sigma_{s}p{>}_{f\lambda }(\eta )=\frac{8f_{vf}}{\pi^{2}}\sum_{i=1}^{N}\frac{x_{i}}{r_{fi}\alpha_{fi}}\int_{0}^{1}\frac{i_{f\lambda }(\eta \cdot \phi )}{\sqrt{(1-\cos\eta )(1+\cos\eta -2\sin^{2}}\phi )}(\sin\phi )$$

where

(9) $$\cos\eta =\mu \mu^{\prime }+{\left[\left(1-\mu^{2})(1-\mu \prime^{2}\right)\right]}^{1/2}\cos(\omega -\omega^{\prime })$$

The scattering cross section–phase function parameter for a parallel slab geometry is obtained by integrating over the azimuthal angle ω as

(10) $$<\sigma_{s}p{>}_{f\lambda }(\mu ,\mu^{\prime })=\frac{1}{2\pi }\int_{0}^{2\pi }<\sigma_{s}p{>}_{f\lambda }(\eta )d\omega$$

Gradated fibrous aerogel composites typically exhibit a fiber volume fraction of less than 5%. According to the independent scattering theory, in such high-porosity fibrous media, independent scattering predominates due to the minimal interaction between fibers. Experimental studies have demonstrated that scattering by aerogel in the infrared region is negligible, and its refractive index approaches unity. Consequently, the radiative properties of the fibrous aerogel composite can be computed by summing the individual contributions of the fibers and the aerogel matrix. The extinction coefficient (K) of the composite is then given by

(11) $$K_{\lambda }=K_{f\lambda }+\sigma_{a\lambda }$$

where $\sigma_{a\lambda }$ is the spectral absorption coefficient of the aerogel. The scattering phase function of the composite remains unaffected by the presence of the aerogel, as its scattering contribution in the infrared is negligible. Theoretical models for the radiative properties of fibrous media with an aerogel matrix have been validated by experimental data [23,69,70]. The accurate prediction of heat transfer through fibrous aerogel composites necessitates a comprehensive consideration of the various heat transfer mechanisms at play. In such dispersed media, the total heat flux comprises contributions from radiation, solid-phase conduction, and gas-phase conduction. The high-porosity structure of aerogels significantly reduces gas-phase convection under atmospheric conditions. Additionally, for low-density aerogels, solid-phase conduction is typically negligible compared to radiative heat transfer at temperatures exceeding 300–400 K [6,42,71,72].

An evaluation of the influence of composite constituents—such as fiber type, size, and volume fraction—on thermal transport can be effectively conducted using the concept of radiative conductivity. The total thermal conductivity can subsequently be estimated by integrating contributions from various heat transfer mechanisms. Detailed analyses of radiative thermal conductivity are extensively covered in the contemporary literature on radiation physics and heat transfer [73]. The current derivation closely follows traditional methodologies, incorporating necessary adjustments to accurately represent the radiative properties of fibers and aerogels. This analysis employs the diffusion approximation for radiative transfer in optically thick, absorbing media, a framework commonly utilized in the study of thermal radiation in porous and fibrous materials [74].

Application of the diffusion approximation yields the radiative heat flux as

(12) $$q_{r}(y)=-\frac{4\pi }{3k_{R}}\frac{dI_{b}(T)}{dy}$$

where $k_{R}$ is typically the Rosseland mean coefficient, which is defined in terms of the spectral extinction coefficient $K_{\lambda }$ by

(13) $$(1/k_{R})=\int_{0}^{\infty }\frac{1}{K_{\lambda }}\frac{dI_{b\lambda }(T)}{dI_{b}(T)}d\lambda$$

The radiative conductivity follows as

(14) $$K_{R}=\frac{16n^{2}\sigma T^{3}}{3k_{R}}$$

and the radiative heat flux is then given by

(15) $$q_{r}(y)=-K_{R}\frac{dT}{dy}$$

To extend the application of the mean extinction coefficient to a scaled transfer coefficient for a medium-containing fiber, a scale factor is applied to $K_{\lambda }$ to account for the scattering-phase function product which gives the transfer coefficient as

(16) $$\Gamma \lambda^{*}=K\lambda (1-\omega \lambda g)$$

where ωλg follows from Equation (10). The scaled coefficient is substituted for Kλ in Equation (11). The total heat transfer by conduction and radiation between two boundaries at temperatures $T_{1}$ and $T_{2}$ is given by

(17) $$q=\frac{1}{C_{0}}\left[\sigma (T_{1}^{4}-T_{2}^{4})+\frac{3K_{c}\tau_{0}}{4L}(T_{1}-T_{2})\right]$$

where

(18) $$C_{0}=\frac{3\tau_{0}}{4}+(\frac{1}{\varepsilon_{1}}-\frac{1}{2})\left[\frac{1}{1+K_{c}/K_{R}(T_{1})}\right]+(\frac{1}{\varepsilon_{2}}-\frac{1}{2})\left[\frac{1}{1+K_{c}/K_{R}(T_{2})}\right]$$

$K_{C}$ is conduction conductivity and the optical depth $\tau_{0}$ is based on the scaled transfer coefficient.

The radiative properties of fiber-reinforced silica aerogel composites were initially computed using Equations (1) through (7). Subsequently, these spectral properties were incorporated into the Radiative Transfer Equation (RTE) to determine the spectral normal transmittances and hemispherical reflectance for various composite thicknesses. Experimental measurements of spectral transmittance and reflectance were conducted for corresponding thicknesses and fiber volume fractions, and the results were compared with the numerical predictions to validate the radiative properties model [75,76,77,78].

Regarding the subject of thermal insulation, choosing the appropriate material for a given application requires an awareness of the temperature range and material qualities of different insulation materials. A thorough classification of various thermal insulation materials, including fiber–aerogel composites and conventional materials, is given in the table below. These materials are grouped according to their structure, temperature range, and important characteristics, all of which are crucial in determining how well they insulate against heat.

Table 1 offers a thorough description of different thermal insulation materials according to their main characteristics, temperature range, and structure. It draws attention to fiber–aerogel composites, which are perfect for sophisticated thermal insulation applications due to their exceptional thermal performance at high temperatures. The table also compares them with traditional materials like mineral wool, fiberglass, and polyurethane foam, emphasizing the difference in their thermal performance in high-temperature environments. For many industrial and residential applications, effective insulation requires careful selection based on heat conductivity and temperature stability.

## 3. Radiative Heat Transfer in Fiber–Aerogel Composites

**Figure 2:**
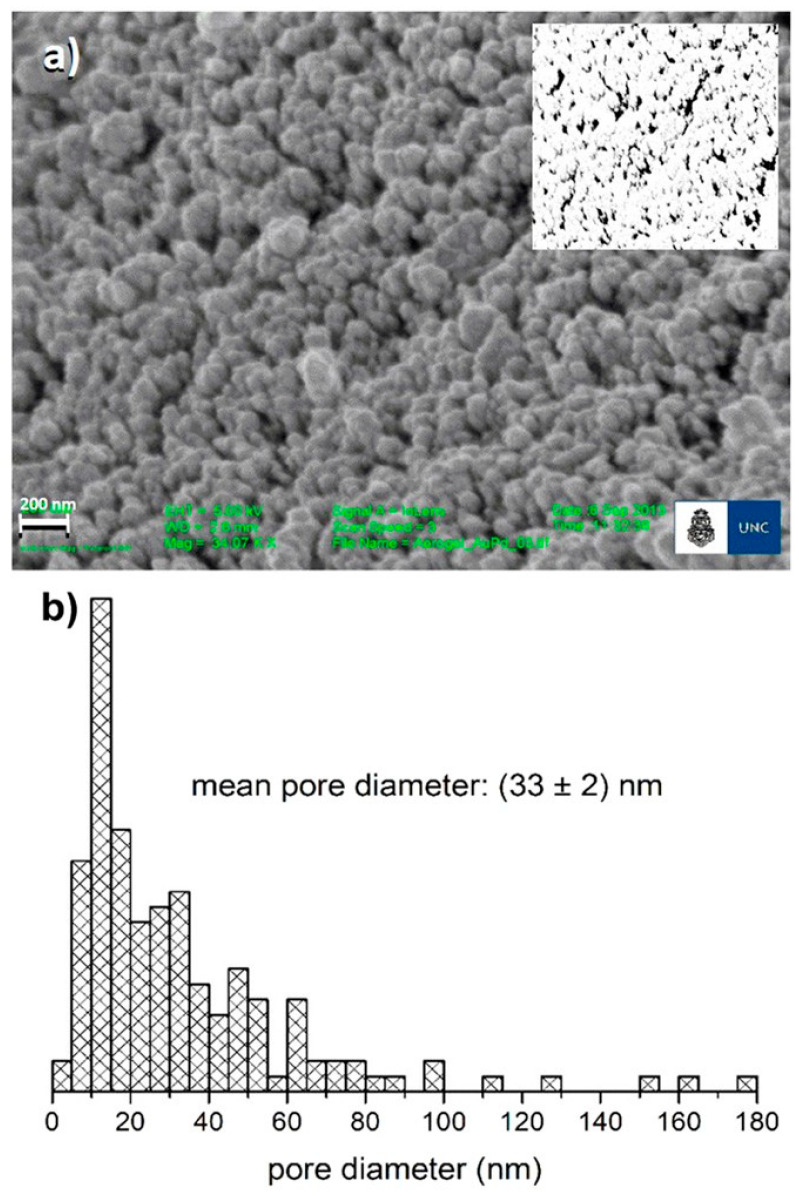
(**a**) SEM image of a bacterial cellulose aerogel prepared via supercritical CO_2_ drying. Experimental procedures are detailed in the Materials and Methods section. The scale bar represents 200 nm. The inset shows the corresponding binary image utilized for porosity analysis. (**b**) Distribution of pore diameters in nm units [57].

Radiative heat transfer within fiber–aerogel composites is profoundly affected by the aerogel’s microstructural parameters, such as porosity, morphological features, and the spatial configuration of embedded fibers. Aerogels are characterized by extremely high porosity (up to 99.8%) and exceptionally low thermal conductivity (typically between 0.01 and 0.06 W/m·K), attributes that arise from their nanoscale pore architecture with an extensively porous network structure, with pore diameters typically spanning from approximately 2 nm to 100 nm. This nanoscale porosity effectively suppresses gaseous heat transfer mechanisms such as conduction and convection, thereby enhancing the overall thermal insulation performance. Nonetheless, at elevated temperatures, the contribution of radiative heat transfer becomes increasingly significant due to the elongation of photon mean free paths within the material. The incorporation of opacifying agents, such as silicon carbide (SiC), into the aerogel matrix serves to markedly attenuate radiative heat transfer by augmenting the material’s extinction coefficient, thus improving its thermal barrier properties. The incorporation of fibers into the aerogel matrix modifies the radiative heat transfer mechanisms by altering both the effective path length and the spatial distribution of thermal radiation traversing the composite material [23,53,89,90,91]. The configuration, morphology, and spatial distribution density of fibers embedded within aerogel composites critically influence the radiative thermal transport properties of the material. Empirical investigations have demonstrated that fibers exhibiting an aspect ratio (length-to-diameter) in the range of 4 to 6 µm, combined with moderate angular inclinations relative to the heat flux direction, effectively enhance the extinction coefficient, thereby diminishing radiative heat transfer. Furthermore, the anisotropic orientation of fibers contributes to directional variations in the composite’s thermal conductivity, significantly impacting the overall thermal transport efficiency [6,54,55,56]. The scanning electron microscopy (SEM) images of the synthesized aerogel revealed a microstructure composed of aggregated particles (Figure 2a). The interstitial voids between these particles, corresponding to pores, displayed a broad size distribution ranging from approximately 3 nm to 180 nm in diameter, as determined by SEM image analysis (Figure 2b). Due to the resolution limitations inherent to SEM, pores smaller than 10 nm may have been underestimated. However, considering that the β-galactosidase enzyme has an approximate diameter of 12–17 nm, pores below this size threshold are unlikely to significantly influence the enzyme’s microenvironment within the aerogel matrix [57].

The accurate prediction of radiative heat transfer within fiber–aerogel composite materials necessitate advanced modeling methodologies. A dual-scale three-dimensional framework, which explicitly simulates the fibrous microstructure and computes the radiative properties of individual fibers, has been implemented to effectively characterize radiative heat transfer phenomena. This approach accounts for the intricate interactions between the fibrous network and the surrounding aerogel matrix, thereby yielding detailed insights into the composite’s thermal transport behavior [42,92]. Temperature fluctuations exert a pronounced influence on radiative heat transfer within aerogel composite materials. Elevated temperatures promote an increase in photon emission rates and extend the mean free path of thermal radiation, thereby intensifying radiative heat transfer mechanisms. In contrast, at reduced temperatures, the lower thermal energy availability results in attenuated radiative contributions. Consequently, the engineering of aerogel composites necessitates the optimization of opacifier concentration and fiber alignment to sustain efficient thermal insulation performance over a broad temperature spectrum [23,45,58,93].

By creatively using numerical simulations for the structural design of fiber aerogels, Liu et al. showed that directionally organized ZrO_2_ fiber aerogels (SZFAs) can significantly lower their high-temperature thermal conductivity by using SiC opacifiers. With a thermal conductivity of just the current W·m^−1^·K^−1^ at 1000 °C, SZFAs produced using the directed freeze-drying process, as anticipated, exhibit significantly better high-temperature thermal insulation capability than current ZrO_2_-based fiber aerogels. SZFAs additionally demonstrate exceptional all-around qualities, such as ultralow density (6.24–37.25 mg·cm^−3^), exceptional elasticity (500 compression cycles at 60% strain), and exceptional heat resistance (up to 1200 °C). The development of SZFAs offers theoretical direction and straightforward building techniques for creating fiber aerogels with superior high-temperature thermal insulation qualities that are utilized in harsh environments.

**Figure 3:**
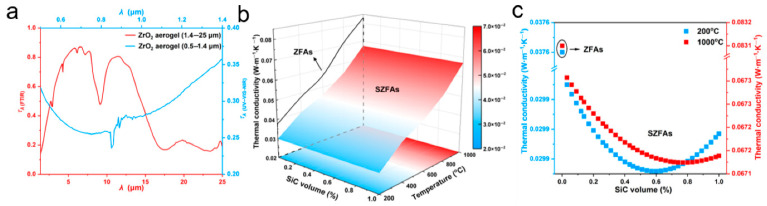
ZFAs’ and SZFAs’ radiative characteristics. (**a**) The ZFAs’ spectral transmittance. (**b**) ZFAs and SZFAs’ thermal conductivity. (**c**) ZFA and SZFA thermal conductivity at 200 and 1000 °C [23].

Figure 3a displays the ideal fiber diameters at different temperatures, and Figure 3b displays the maximum temperature-dependent mean extinction coefficient of fibers based on the ideal diameter. The findings indicate that ZrO_2_ fibers outperform the other two fibers in terms of extinction over the entire temperature range of 200–1000 °C. Additionally, the ideal diameter of ZrO_2_ fibers steadily decreases as the temperature rises, from 2.6 μm at 200 °C to 1.1 μm at 1000 °C. Consequently, ZrO_2_ fibers with an average diameter of 1 μm were selected as the fundamental unit for creating fiber aerogels in consideration of the high-temperature application situation of aerogels [23].

Significant anisotropy exists in the fiber, and both radiative thermal conductivity (*k_r_*) and conductive thermal conductivity (*k_c_*) are strongly influenced by its orientation direction. Therefore, the effects of fiber alignment on fiber aerogel shading performance were initially examined. The radiation properties of ZrO_2_ fibers (1 μm diameter) at different angles between the fiber axis and incident radiation were assessed using Mie scattering theory (Figure 3c). The findings show that when the fiber axis is perpendicular to the incident radiation, or the incidence angle is 90°, ZrO_2_ fibers have the highest temperature-dependent mean extinction coefficient [23].

## 4. Factors Affecting Radiative Heat Transfer Properties

**Figure 4:**
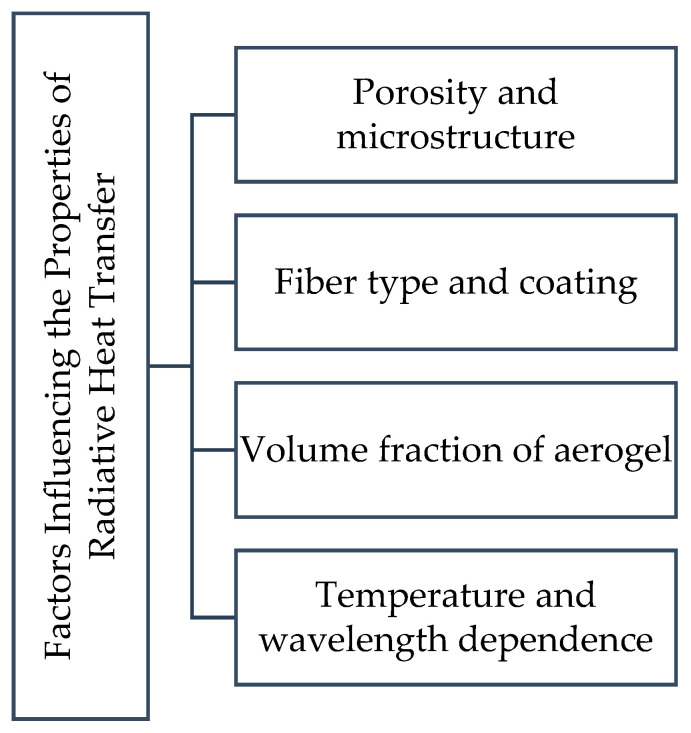
Factors Influencing the Properties of Radiative Heat Transfer.

The radiative heat transfer characteristics of fiber–aerogel composites—which include the emission, absorption, and scattering of thermal radiation—have a significant impact on how well they perform in thermal insulation. These characteristics are greatly influenced by important variables, including the aerogel’s volume percentage and its reliance on temperature and wavelength. Optimizing the design of composites for different applications requires an understanding of these aspects. This information enhances the composite’s ability to insulate against a range of environmental factors, including high temperatures and variable radiation levels. Researchers can improve fiber–aerogel composites’ mechanical and thermal performance in sectors including energy-efficient systems, construction, and aerospace by looking into these variables. In Figure 4, factors influencing the properties of radiative heat transfer are given and explained in detail.

The intricate interaction of the material and its microstructural characteristics controls radiative heat transmission in fibrous composites. Because finer fibers increase scattering and decrease transmittance, fiber diameter is a crucial element that inversely corresponds with radiative heat flux at constant porosity. Optical route length and extinction efficiency are influenced by fiber orientation and packing density (porosity); greater packing fractions reduce radiative transmittance. Fiber surface emissivity and absorptivity have a direct impact on the dynamics of energy absorption and emission, higher emissivity results in less radiation penetration. Furthermore, wavelength-dependent scattering and absorption behavior are determined by material optical properties, such as refractive index and absorption coefficients, which must be optimized for certain thermal settings. By adding spectrum selectivity and interfacial reflections, multilayer structures and opacifiers further modify radiative transmission. Together, these factors determine how well the composite works in a variety of applications, from high-temperature industrial systems to aerospace insulation [94,95].

In fibrous composites, porosity has a major effect on radiative heat transport. Because of the huge volume of air pockets that serve as thermal insulators, high porosity materials—like aerogels—have poor thermal conductivity. For example, thermal conductivities as low as 0.013 W/(m·K) are seen in carbon aerogels with over 50% porosity and pore sizes less than 100 nm [80]. Another important factor is the microstructure, which includes the distribution of pore sizes and the arrangement of fibers. According to research, randomly oriented fiber assemblies have lower radiative heat conductivity than parallel arrangements, and the radiative heat conductivity decreases with bulk density [96,97].

The radiative properties of fibers are greatly influenced by their surface coatings and material composition. Glass fibers are excellent insulators due to their low thermal conductivity, which is around 0.05 W/(m·K). Heat treatment and surface coatings can modify the infrared emissivity of carbon fibers. For instance, because of their distinct structural characteristics, carbon nanotube (CNT) coatings can raise emissivity while pyrolytic carbon (PyC) coatings can decrease it. Reflectivity can be increased, and radiative heat transfer can be decreased by applying dielectric films, such as TiO_2_ or TiO_2_/SiO_2_ multilayer coatings, to fibers. In order to attain high reflectivity at particular wavelengths, these coatings take advantage of constructive interference [98,99,100,101].

The thermal insulation qualities of fiber–aerogel composites are greatly influenced by the aerogel volume fraction, or Vf. Because of the aerogel’s high porosity and low thermal conductivity, increasing Vf typically improves the material’s capacity to lower thermal conductivity. Fibers can, however, contribute new heat transfer pathways, such as solid-phase conduction via the fibers and aerogel framework, gas-phase conduction between aerogel pores, and thermal radiation within the composite, even though they increase mechanical strength [6,102]. According to a study by Yang et al. (2015), the effective thermal conductivity of silica aerogel composites decreases as the fiber volume percentage increases. This decrease is explained by the fact that as the fiber content increases, the cross-sectional area available for heat conduction decreases and the tortuosity increases. The thermal conductivities of silica aerogel composite insulating materials were calculated using a combined heat conduction and radiation model. The results show that as the mass percentage of additive increases, the overall conductivity initially falls and subsequently increases [32,45,103]. Temperature and thermal radiation wavelength have an impact on radiative heat transfer in fiber–aerogel composites. Higher radiative thermal conductivities may result from increasing infrared radiation emission at higher temperatures. In the 2–8 μm region, where pure silica aerogels have high transmittance and cause substantial radiative heat transfer, the wavelength dependence is most noticeable. This phenomenon results from silica aerogels’ infrared transparency in this spectral region, which permits greater heat radiation to flow through the substance [90,104]. This can be lessened by lowering radiative heat conductivity through the use of opacifiers like SiC nanoparticles. For example, Zhang et al. (2023) demonstrated the efficiency of opacifiers in reducing radiative heat transfer by finding that adding 4% volume fraction SiC nanowires to silica aerogel composites reduced the thermal conductivity at 500 °C to 0.040 W/(m·K) [23,45,105,106].

The aerogel volume percentage in fiber–aerogel composites is a key factor influencing the material’s ability to insulate against heat, especially when it comes to radiative heat transmission. The overall porosity of the composite rises with an increase in the volume percentage of aerogel, which usually leads to improved thermal insulation because less conductive heat transfer occurs. Aerogels effectively stop heat flow in insulating applications because of their exceptionally low thermal conductivity, which is mostly due to their high porosity and low density.

However, the behavior of radiative heat transmission is more intricate and heavily impacted by the optical characteristics of the aerogel. The composite may show improved radiative scattering and absorption at greater aerogel fractions, which helps to lessen heat transfer by radiation. Aerogels’ optical thickness—the ratio of the material’s thickness to the radiation’s mean free path—is the primary determinant of radiation heat transfer. The type of fiber and its packing density in composites also affect how well the aerogel material scatters and absorbs light. The radiative conductivity of the composite has been shown to rise with an increase in the volume fraction of aerogel; however, this improvement reaches a saturation point. After this, the fiber network limits the composite’s capacity to scatter and absorb radiation; thus, additional increases in the aerogel volume fraction only slightly improve radiative heat transfer. Optimizing thermal insulation requires striking a compromise between the mechanical and thermal contributions of the fiber and the optical qualities of the aerogel [6,107].

**Figure 5:**
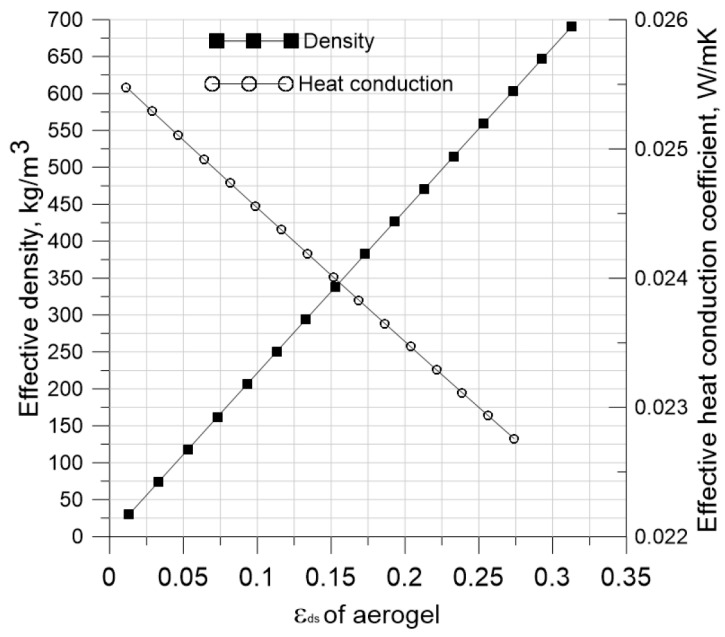
Effects of the aerogel volume fraction *εds* on the effective density and coefficient of thermal conductivity in the middle of the clothing’s batting (insulating layer) [107].

Since the effective thermal conductivity drops and thermal equilibrium is reached over extended periods of time, the beginning conditions are crucial for studying aerogel materials with a large volume fraction of fibers *εds*. Consequently, the fibers’ volume fraction *εds* rises, increasing the material’s effective density and decreasing its effective coefficient of heat conductivity (Figure 5). In technical terms, using aerogel particles as the infill material inside a polymer matrix made up of thick monofilament polymer fibers with a diameter of 100–200 μ increases the aerogel fraction [82,107,108].

Temperature and radiation wavelength have a substantial impact on the radiative heat transfer in fiber–aerogel composites. Radiative heat transmission is directly impacted by changes in the material’s emissivity, or capacity to emit thermal radiation, as the temperature rises. For use in environments with changing temperatures or varied radiation spectra, like in aerospace or high-temperature industrial operations, the wavelength of the incident radiation determines the spectrum absorption and emission characteristics of aerogels [32].

Aerogels often become opaquer to infrared light at high temperatures, particularly when combined with infrared opacifiers like SiC or TiO_2_, which improve the material’s absorption and dispersion of infrared light. The Stefan–Boltzmann law, which asserts that radiative heat transmission rises with the fourth power of temperature, can be used to characterize the relationship between temperature and radiative heat transfer. This suggests that for fiber–aerogel composites, temperature influences both the optical characteristics of the fiber and aerogel components, especially at the longer infrared wavelengths, in addition to emissivity [82].

Furthermore, establishing effective thermal insulation requires consideration of the wavelength dependency of radiative heat transmission. Aerogels are typically transparent at short wavelengths (visible light and near-infrared), but they grow increasingly opaque at longer wavelengths (infrared). The radiative conductivity of the composite is therefore impacted by variations in the radiation spectrum, for as when switching from solar light (short wavelengths) to heat radiation (long wavelengths). Because of this, the material can be used in applications that need thermal insulation against longwave and shortwave solar radiation [109].

**Figure 6:**
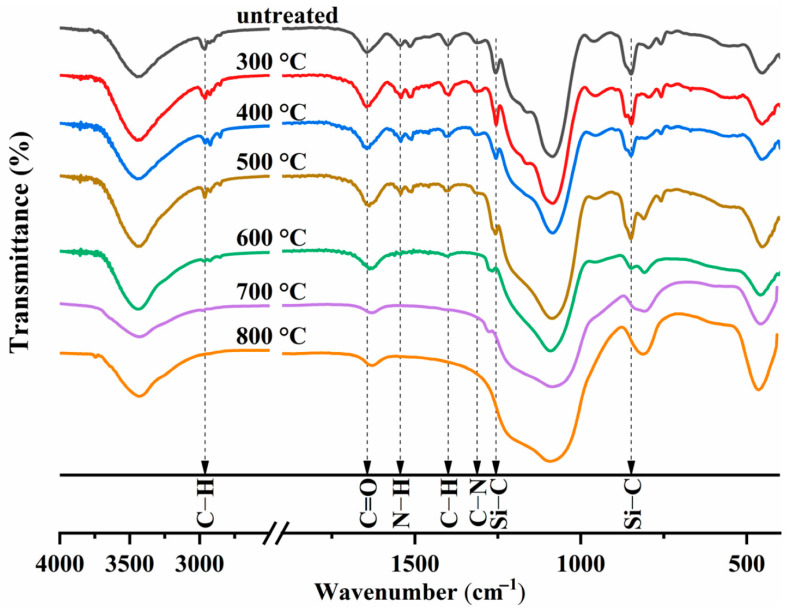
AP/aerogel FTIR spectra following heat treatment at various temperatures [112].

Figure 6 shows an analysis of how the heat treatment temperature affects the chemical bonds and distinctive functional groups. The AP/aerogel’s IR spectra show no discernible change at temperatures lower than 500 °C. While the Si–C bonds at 1256 cm^−1^ shift to 1268 cm^−1^, indicating the thermal decomposition of Si–(CH_3_)_3_ groups, the N–H, C–N, and C=O bonds vanish when the heat treatment temperature reaches 600 °C, suggesting that the amide groups are pyrolyzed between 500 and 600 °C [109,110]. The intensities of the Si–C (1268 cm^−1^ and 848 cm^−1^) and C–H (2963 cm^−1^) bonds progressively diminish as the heat treatment temperature rises, and they ultimately vanish at 800 °C. These modifications to chemical bonds confirm the decrease in methyl groups, which has a major impact on the AP/aerogel’s hydrophobicity [111].

**Figure 7:**
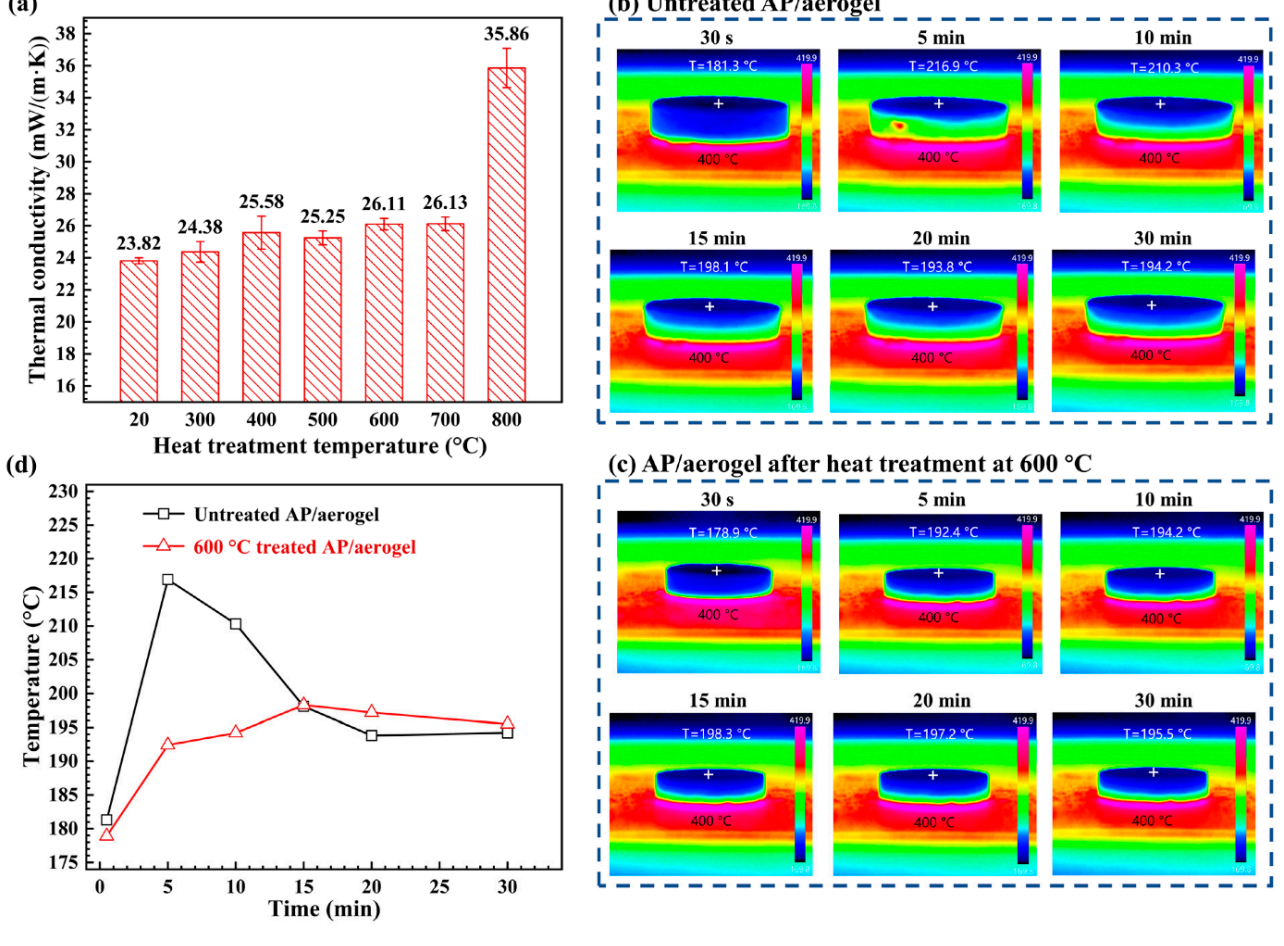
(**a**) Infrared thermographic pictures; (**b**,**c**) thermal conductivities of the AP/aerogels at various heat treatment temperatures; and (**d**) the upper surface temperature of the AP/aerogels both untreated and treated at 600 °C [112].

The thermal conductivities of the AP/aerogels at various heat treatment temperatures are displayed in Figure 7a. The thermal conductivity of the untreated AP/aerogel is 23.82 mW/m/K. The thermal conductivity rises marginally to 26.13 mW/m/K when the heat treatment temperature reaches 700 °C, but it is still quite near that of static air. The thermal conductivity sharply rises to 35.86 mW/m/K upon heat treatment at 800 °C, suggesting that the AP/aerogel’s thermal insulation ability sharply declines. The evolution of microstructures during heat treatment is clearly the reason for the increase in thermal conductivity [112]. The surface temperature profiles of untreated and heat-treated AP/aerogels placed on a 400 °C hot plate are shown in Figure 7b,c, while Figure 7d shows the upper surface temperatures. After five minutes, the untreated AP/aerogel reached a high temperature of 216.9 °C, and after thirty minutes, it progressively cooled to 194.2 °C. However, after being treated at 600 °C, the AP/aerogel rose to 192.4 °C after 5 min, reached 198.3 °C after 15 min, and finally settled at 195.5 °C after 30 min. The thermal breakdown of silyl methyl groups (Si–(CH_3_)_3_) in the silica structure releases heat that intensifies the temperature rise, causing the untreated AP/aerogel to exhibit a strong peak early on and a rapid temperature rise. Thermal shock or even fire hazards could result from this. The heat-treated AP/aerogels, on the other hand, undergo a more steady and gradual temperature rise, lowering the possibility of thermal instability and danger.

All things considered, heat-treated AP/aerogels below 700 °C maintain their low thermal conductivity without noticeably deteriorating their insulating capabilities. Furthermore, heat treatment supports the wider use of silica aerogels in thermal insulation by preventing heat shock and related hazards [112].

## 5. Experimental Studies and Results

Optimizing the efficacy of thermal insulation requires an understanding of the radiative heat transfer characteristics of materials. Important characteristics that affect a material’s ability to insulate against heat radiation are emissivity, transmittance, and reflectance. Radiative heat transmission in fiber–aerogel composites is evaluated using methods such as high-temperature emissivity testing, integrating sphere measurements, and FTIR spectroscopy. These techniques provide information on the thermal performance of aerogels and fibers by determining how they interact with radiation.

Because of their mesoporous structure, which lowers heat transfer by conduction, convection, and radiation, fiber–aerogel composites are renowned for having low radiative heat transfer. Aerogels and fibers can be combined to increase mechanical strength without sacrificing thermal characteristics. Their performance is affected by variables such as temperature, aerogel porosity, and fiber orientation.

Fiber–aerogel composites have better radiative heat transfer qualities than conventional materials like mineral wool, fiberglass, and foam, which makes them perfect for high-performance uses in high-temperature industries, energy-efficient buildings, and aerospace [113,114].

**Figure 8:**
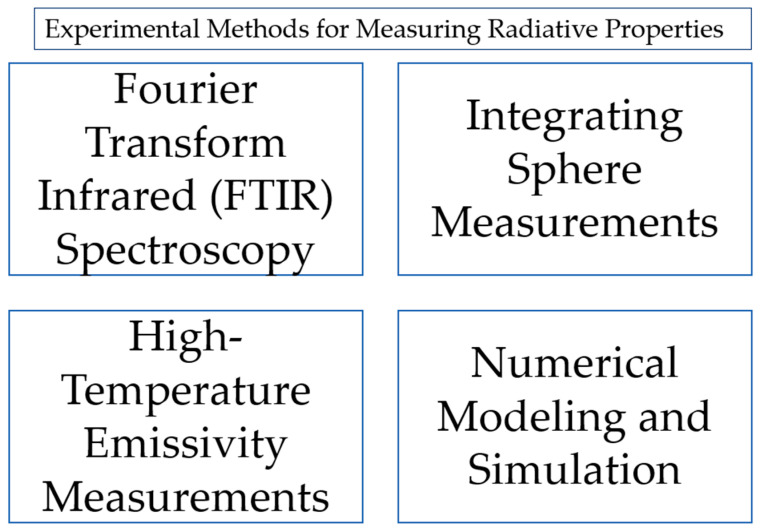
Techniques for Measuring Radiative Properties Experimentally.

As shown in Figure 8, FTIR spectroscopy is one of the methods frequently employed to assess the spectrum transmittance and reflectance of fiber–aerogel composites. The extinction coefficient, which is crucial for figuring out radiative heat conductivity, is found by examining the absorption spectra.

The heat radiative transmission in silica aerogel and silica aerogel composite insulation materials (a ceramic fiber–aerogel composite and a xonotlite–aerogel composite) was investigated by Wei et al. Using a Fourier transform infrared spectrometer (FTIR), the spectral transmittances of ceramic fiber insulation materials, silica aerogel, and xonotlite-type calcium silicate—all semi-transparent materials that can absorb, emit, and scatter thermal radiation—are measured at various infrared wavelengths between 2.5 and 25 μm at room temperature. Each sample’s specific Rosseland mean extinction coefficient and specific spectral extinction coefficient are ascertained using the spectral transmittances [32]. Wang et al. demonstrated continuous, hierarchically porous silk fibroin/graphene oxide (SF/GO) aerogel fibers with superior radiative heating, thermal insulation, and strength. The coaxial wet spinning and freeze-drying methods were used to create these fibers. Multiscale porous structures are present in the hollow CA/PAA fibers, which are made of cellulose acetate/poly (acrylic acid) and are produced by wet spinning coaxial. These structures improve the mechanical strength of the aerogel fibers in addition to helping to produce the SF/GO aerogel core. There are a number of reasons why the hollow CA/PAA, SF, and SF/GO aerogel fibers exhibit radiative heating properties: (i) the hollow CA/PAA fibers’ porous sheath absorbs very little infrared radiation; the remainder is reflected onto the fiber surface, raising the temperature of the fiber surface; (ii) the SF aerogel inside the composite fibers’ core absorbs some infrared radiation, increasing the reflected energy and raising the temperature of the SF aerogel fibers even more. Numerous investigations have shown that the graphene sheets contained in the SF/GO aerogel fibers are very good at collecting infrared energy from outside radiation. When subjected to infrared radiation, the SF/GO 2.0 aerogel fibers demonstrated the best radiative heating capability because of their superior infrared absorption and infrared energy reflection onto the fiber surface [115].

**Figure 9:**
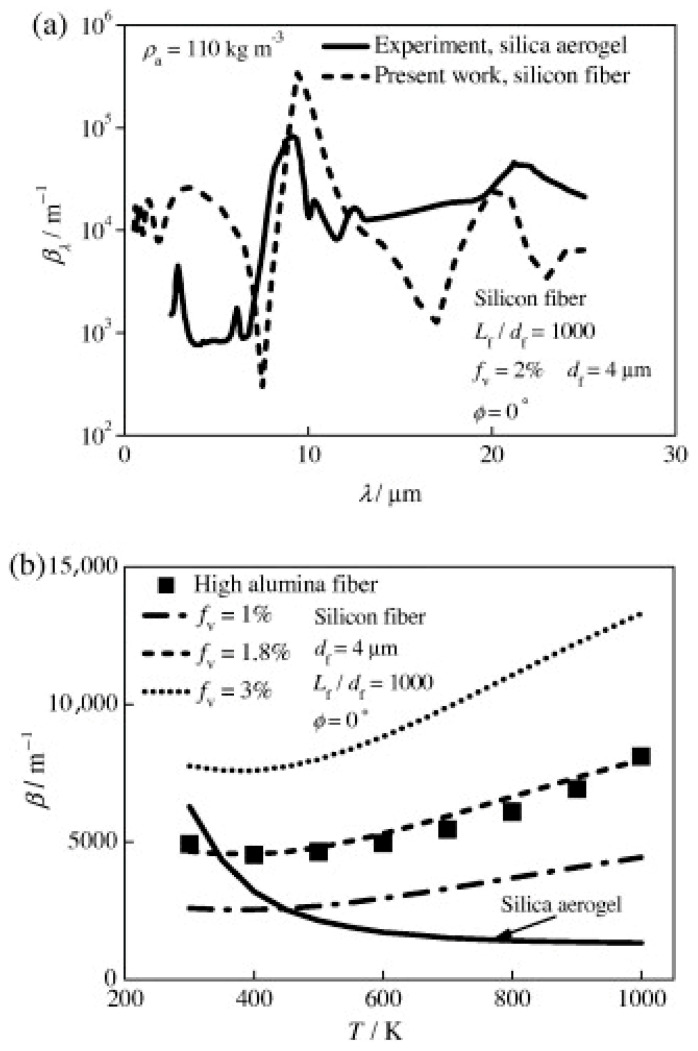
Fiber and silica aerogel extinction coefficients, Rosseland mean extinction coefficients (**a**), and spectral extinction coefficients (**b**) [6].

Zhao et al. used a modified anomalous diffraction theory in a combined heat conduction and radiation model to investigate the radiative characteristics and heat transmission in fiber-loaded silica aerogel composites. To faithfully model the structure of the material, a 2-D fiber distribution with random parameters was created. The effective thermal conductivity of the composite was calculated by using the finite volume method to solve the steady-state energy equation and the two-flux radiation model. For the purpose of optimizing material characteristics like the inclination angle, fiber diameter, and length-to-diameter ratio, the numerical findings offer theoretical insights. The modified ADT (anomalous diffraction theory) produced findings within 10% of the Mie theory accuracy for the spectral and Rosseland mean extinction coefficients of the fibers, βfλ and βf, as shown in Figure 9a,b [116]. Based on the measured spectral extinction coefficient, βaλ, acquired from FTIR measurements with an accuracy of within 5% [29], the Rosseland extinction coefficient of the silica aerogel, βa, displayed in Figure 9a, was determined from Equation (19).

(19) $$\frac{1}{\beta (T)}=\int_{0}^{\infty }\frac{1}{\beta_{\lambda }}*\frac{{\partial E}_{b\lambda }}{{\partial E}_{b}}d\lambda \approx \int_{\lambda_{1}}^{\lambda_{2}}\frac{1}{\beta_{\lambda }}*\frac{{\partial E}_{b\lambda }}{{\partial E}_{b}}d\lambda$$

For high alumina fibers at fv = 5% and df = 1–10 μm, Figure 9b shows that the fiber extinction coefficients predicted by the modified ADT fall within the same range as experimental data [117]. The fiber extinction coefficient rises in tandem with the fiber volume fraction. Furthermore, at higher temperatures, the silica aerogel’s extinction coefficient is much lower than the fiber’s, decreasing with temperature. This demonstrates how fiber greatly increases the composite’s effective extinction coefficient, which aids in lowering radiative heat transfer at high temperatures [6].

**Figure 10:**
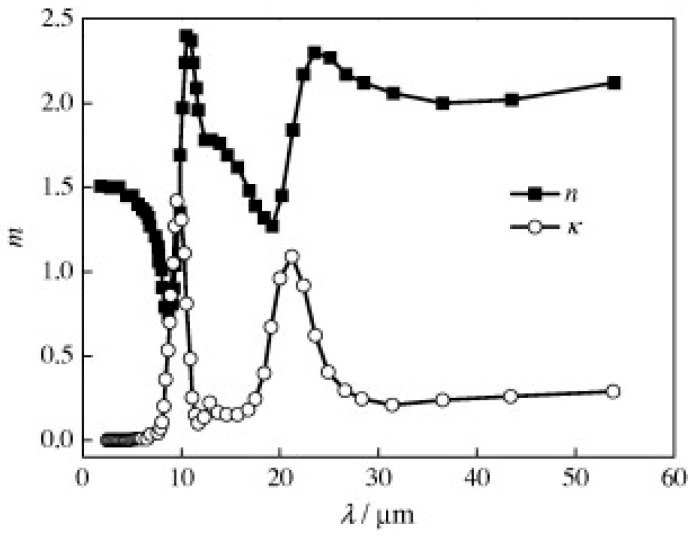
Refractive index of the silicon fiber complex [6].

**Figure 11:**
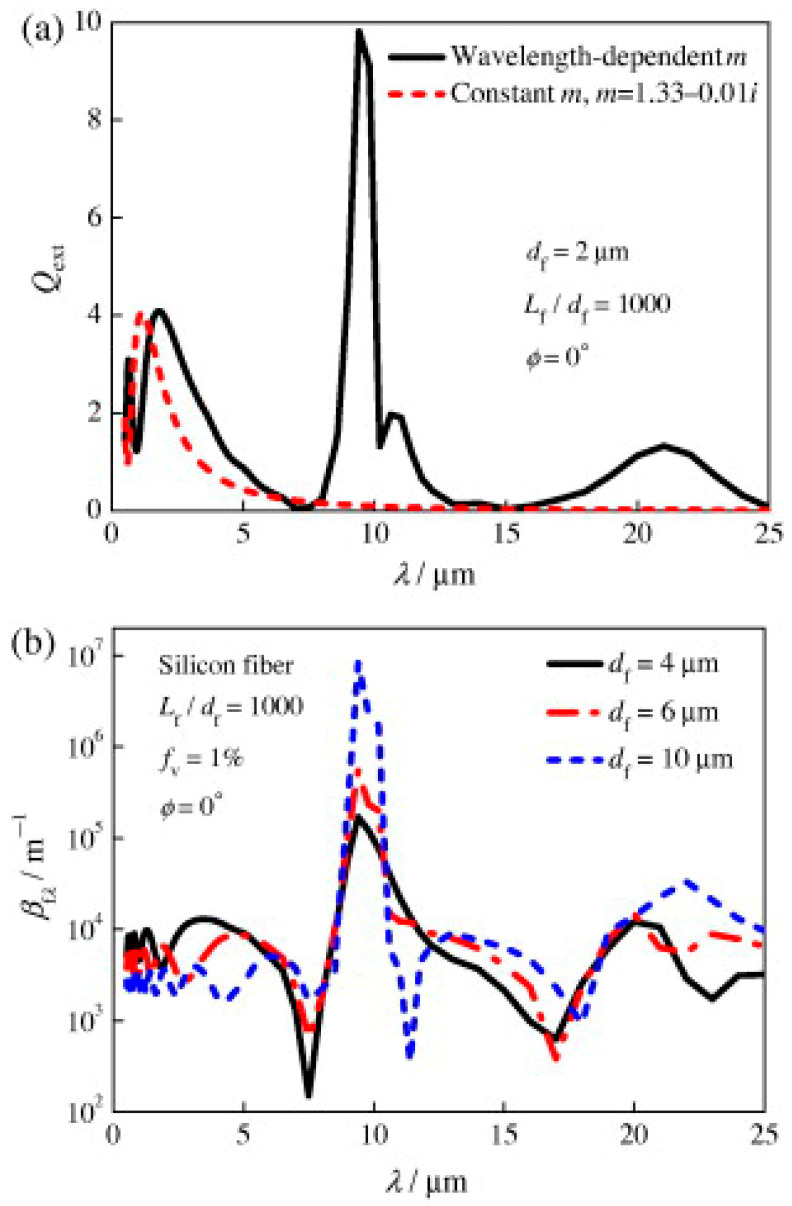
Radiative properties of fibers: (**a**) spectrum extinction coefficient for different diameters; (**b**) extinction efficiency for constant- and wavelength-dependent complex refractive indices [6].

Using the spectrum complex refractive index in Figure 10 and Figure 11a demonstrates that the fiber extinction efficiency peaks at around 9.5 μm. However, for λ > 5 μm, the fiber extinction efficiency falls with increasing wavelength for the constant complex refractive index scenario, m = 1.33−0.01i. For λ > 5 μm, the fiber extinction efficiency is therefore typically significantly underestimated when the assumption of constant fiber optical characteristics is used.

Figure 11b demonstrates that the fiber diameter, df, has a considerable impact on the spectrum extinction coefficient of fibers, βfλ. While the average of βfλ for wavelengths of 0.5−5 μm drops with df, the peak for βfλ at λ = 9−9.4 μm grows with df.

**Figure 12:**
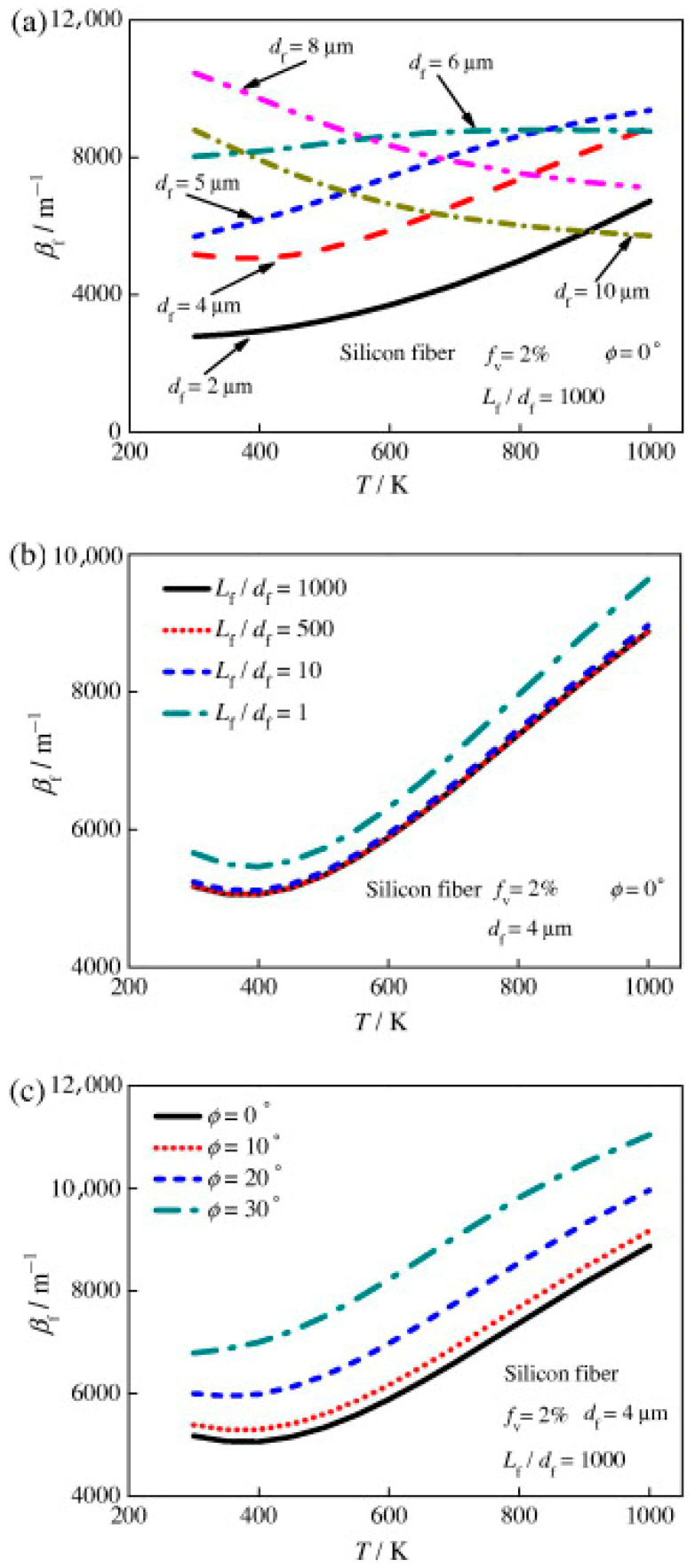
Extinction coefficient for fibers: (**a**) for different diameters; (**b**) for different ratios of length to diameter; and (**c**) for different angles of inclination [6].

Thus, Figure 12a demonstrates that the Rosseland mean extinction coefficient of the fibers, βf, falls with temperature for 8−10 μm diameter fibers and increases with temperature for 2−5 μm diameter fibers after 400 K. The blue shift indicated by Planck’s law and Equation (19), as well as the fluctuation of βfλ with df (Figure 11b), are the causes of the significant variations in βf for different fiber diameters. In order to maximize the extinction coefficient, the 6−8 μm diameter fibers should be positioned adjacent to the cold side, whereas the 5−6 μm diameter fibers should be positioned near the hot side for T > 600 K [6].

**Figure 13:**
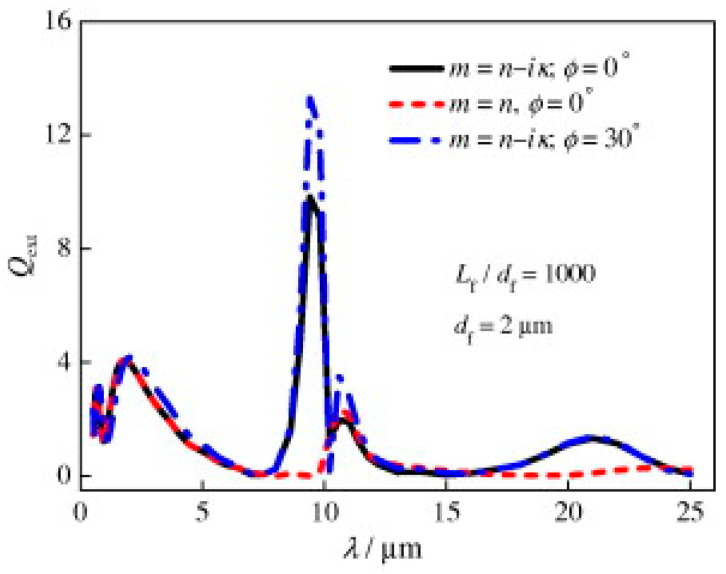
The impact of the fiber extinction coefficient on the imaginary component of the complex refractive index [6].

In qualitative agreement with experimental observations, Figure 12b demonstrates that βf increases when the fiber length-to-diameter ratio, Lf/df, decreases [34,105]. In Equation (20), Lf/df was incorporated into the edge effect term, Qedge. Changes in the fiber extinction coefficient for Lf/df = 500−1000 are negligible. As the inclination angle, ϕ, increases, βf also increases, as seen in Figure 12c. Figure 13 illustrates that the spectral fiber extinction efficiency for ϕ = 30° increases more around λ = 9.5 μm compared to other wavelengths, primarily due to the influence of the imaginary component of the complex refractive index, κ [6].

(20) Qext,large=Qad+Qad2Qedge +1[c1Qad+1]

Because Lind and Greenberg [118] assumed a constant complex refractive index, m = 1.6, and ignored the influence of the imaginary component of the index, they discovered that βf decreases as the inclination angle increases. As a result, as Figure 13 illustrates, they significantly underestimated the extinction coefficient for λ > 8 μm and ignored the fiber absorption impact [6].

The goal of recent studies has been to manipulate the radiative heat transfer characteristics of fiber–aerogel composites in order to improve their thermal insulation qualities. Key radiative characteristics including emissivity, reflectance, and transmittance are measured in these investigations using a variety of experimental approaches. The effect of fiber content and orientation on the radiative characteristics of fiber–aerogel composites was examined by Liu et al. in 2023 [23]. They discovered that adding components like SiC as opacifiers and orienting fibers in a certain direction greatly decreased radiative heat transmission, improving the composites’ overall thermal insulation efficiency. At 1000 °C, this method showed a decrease in thermal conductivity to 0.0663 W·m^−1^·K^−1^, suggesting enhanced high-temperature insulation properties [23]. A novel SiO_2_-ZrO_2_ fiber aerogel–TiO_2_ composite (SZTFAs) was created by Han et al. (2025) to address the significant radiative heat transfer found in conventional fiber aerogels. They improved thermal insulation qualities by successfully lowering infrared transmission by applying TiO_2_ nanolayers to the fiber surfaces. Extreme heat conditions can be tolerated by the SZTFAs due to their great mechanical strength, low thermal conductivity (0.0292 W·m^−1^·K^−1^), and exceptional high-temperature stability [119]. A study on aerogel blankets with different porosities and fiber compositions was carried out by Hoseini et al. (2016). According to their research, better thermal conductivity resulted from improved solid and gas conduction pathways caused by increasing fiber content and lowering porosity. These modifications, however, also had an impact on radiative heat transport, indicating a complicated relationship between thermal performance and structural characteristics [120].

As seen in Table 2 Comparing the Properties of Radiative Heat Transfer, fiber–aerogel composites offer excellent thermal performance by successfully lowering both conductive and radiative heat transfer, making them an appealing substitute for conventional insulation materials. Together with improvements in material processing and design, their improved insulating qualities make them a viable option for high-temperature industrial operations, energy-efficient buildings, and aerospace applications that demand great thermal resistance.

A summary of the main conclusions, approaches, difficulties, and potential avenues for further study regarding radiative heat transfer in fiber–aerogel composites is given in Table 3. It draws attention to the noteworthy contributions made by different studies and points out crucial areas that require more investigation in order to maximize the performance of these composites in high-temperature and energy-efficient applications.

Furthermore, recent experimental research has investigated a number of scalable manufacturing processes for creating fiber–aerogel composites, with an emphasis on both industrial viability and thermal insulation efficacy. The material’s mechanical strength, thermal conductivity, and general performance have all improved thanks to these manufacturing techniques, opening the door for wider industrial applications. One such technique is ambient pressure drying (APD), which lowers manufacturing costs and energy consumption by doing away with the requirement for high-pressure supercritical drying. It has been demonstrated that this method maintains the low heat conductivity of the aerogel while increasing its mechanical flexibility, particularly when paired with cellulose fibers for increased strength and thermal insulation capabilities [138]. Roll-to-roll additive manufacturing is another noteworthy method that makes large-area fabrication more affordable by enabling the continuous manufacture of aerogel composites. According to experimental findings, aerogels made with this technique have superior compressive strength and thermal resistance (high R-values), which makes them perfect for use in building insulation applications [139]. Furthermore, to improve the mechanical strength and thermal insulation qualities of the composite, fibrous aerogels with regulated porosity and alignment are produced by electrospinning, especially wet electrospinning. Because of their enhanced compressive strength and thermal conductivity, these electrospun fibers can be used for structural purposes in a variety of industries [140,141]. All things considered, it has been established that these scalable manufacturing processes yield fiber–aerogel composites with thermal performance that is on par with or better than that of conventional approaches, allowing for their wider use in sectors including automotive, aircraft, and construction. To fully realize these materials’ potential in high-volume industrial applications, however, issues with cost, material availability, and uniformity must still be resolved [142].

## 6. Applications for Fiber–Aerogel Composites

Fiber–aerogel composites have drawn a lot of interest because of their exceptional mechanical strength, low density, and thermal insulation qualities. The advantages of aerogels, which are renowned for having very low heat conductivity, are combined in these composites with the additional flexibility and structural support of fibrous materials. Fiber–aerogel composites are therefore being investigated more and more for a variety of uses in sectors where weight reduction, energy efficiency, and thermal insulation are essential.

These composites have demonstrated their adaptability in a variety of applications, ranging from building materials intended to increase energy efficiency in construction to the aerospace and automobile sectors that require superior thermal protection. They are used in solar energy systems, industrial furnaces, and cryogenics, where they minimize heat loss and maximize energy use. Furthermore, the future potential of fiber–aerogel composites is highlighted by new applications in wearable thermal insulation and next-generation energy-efficient systems. The various uses of these composites will be discussed in this part, along with their place in contemporary technologies and potential for further advancements.

*Construction and Building*

Because of their remarkable thermal insulating qualities, high mechanical strength, and lightweight nature, fiber–aerogel composites are being utilized more and more in the building and construction sector. These composites are especially useful for applications like insulating materials for walls, ceilings, and roofs that call for space savings and thermal energy efficiency. Fiber–aerogel composites are perfect for energy-efficient structures that require high-performance insulation without adding undue weight or bulk because of their lightweight nature and capacity to decrease heat transmission [143].

According to recent studies, insulation materials based on fibrous aerogel can significantly lower thermal conductivity to 0.015 W/m·K at room temperature, outperforming more conventional materials like mineral wool and fiberglass. The excellent thermal resistance of the aerogel fibers helps to improve building climate control and energy savings. Because it can cut carbon footprints by reducing the energy needed for building heating and cooling, this material also promotes sustainability [144,145].

Nocentini et al. investigated the thermal performance of composite materials known as “aerogel blankets”, which consist of silica aerogel embedded within a fibrous inorganic mat. An experimentally validated mathematical model is developed to predict the effective thermal conductivity of these blankets as a function of density, fiber volume fraction, and temperature. Experimental validation is conducted using a test building retrofitted with a needle glass fiber (NGF) aerogel blanket, with thermal measurements compared to the pre-retrofit baseline. The U-value of the retrofitted wall is determined using both stationary and transient methods. Additionally, a whole-building Energy Plus simulation predicts annual energy consumption for a detached house retrofitted with the NGF aerogel blanket in various European climates. Economic analysis is performed using a 1-D COMSOL model to assess profitability.

Results indicated that aerogel blankets exhibit a low effective thermal conductivity (~0.0165 W·m^−1^·K^−1^). The mathematical model accurately predicts gaseous conduction, though discrepancies arise for radiative and solid conduction components. The effective thermal conductivity increases with fiber volume fraction and temperature, and shows a concave dependence on density, with a minimum at approximately 140 kg·m^−3^. Retrofitting with a 2.5 cm NGF aerogel blanket nearly doubles wall thermal resistance and reduces heating loads by over 40% across all tested climates. Economic analysis suggests that aerogel blankets are particularly cost-effective in areas with high floor area prices, as they provide superior insulation without significantly reducing usable indoor space [130].

*The automotive and aerospace industries*

Thermal insulation is essential in the automotive and aerospace sectors, especially in settings subjected to high temperatures. Fiber–aerogel composites offer exceptional thermal protection for uses like car exhaust systems, jet engine parts, and spacecraft insulation. These composites are perfect for thermal protection systems (TPS) because they retain low thermal conductivity even in harsh environments.

The lightweight properties of fiber–aerogel composites and their resistance to high temperatures (up to 1000 °C) make them ideal for space exploration and aircraft propulsion systems. Aerogels are utilized in the automotive industry to insulate exhaust systems because they provide fire protection and thermal insulation without significantly increasing weight. Since the automotive and aerospace industries are concerned with enhancing safety and fuel efficiency, the incorporation of fiber–aerogel composites allows for more efficient temperature control without adding weight to individual components [82,108].

*Energy-Efficient Technologies*

Fiber–aerogel composites are essential to energy-saving technologies in a range of industrial settings. Cryogenics is one of the most prominent applications, where aerogels offer the best insulation in low-temperature settings like cryogenic storage containers and liquid natural gas (LNG) tanks. In cryogenic temperatures, these composites can sustain incredibly low thermal conductivity, avoiding energy loss during transportation and storage of liquid gases.

Fiber–aerogel composites are utilized in high-temperature reactors and industrial furnaces to reduce heat loss and increase energy efficiency. These substances are also necessary for solar energy systems because they insulate solar thermal collectors, which keep the system’s temperature high. More effective thermal storage and energy transfer are made possible by these composites’ low thermal conductivity and lightweight nature, which are essential for sustainable energy systems [146,147].

*New and Developing Uses*

New uses for fiber–aerogel composites are anticipated as studies continue to examine their possibilities. Wearable thermal insulation is one example of this. Because they are incredibly light and effective at insulating, aerogels are being researched for use in protective gear and apparel. Without sacrificing comfort or mobility, these materials can offer thermal protection in harsh settings, such as space missions or military activities.

Furthermore, it is projected that the incorporation of fiber–aerogel composites into next-generation energy-efficient systems will greatly enhance thermal management in heat exchangers, solar panels, and smart houses. Fiber–aerogel composites are a promising material for future energy systems because of their lightweight, robust, and efficient thermal qualities, which allow for improved thermal storage, energy harvesting, and temperature management [148,149].

Commercial solutions based on fiber–aerogel composites are currently offered in a number of industries, especially for thermal insulation purposes. Leading businesses have created consumer and industrial insulation products based on aerogel. In order to improve mechanical strength, flexibility, and thermal stability while reducing heat transfer, these products frequently blend aerogels with fibers or textiles. Aspen Aerogels (Pyrogel^®^, Northborough, MA, USA), (Thermablok^®^, Hopkinton, MA, USA), and Aerogel Blanket Insulation (Aerogel Technologie, Hopkinton, MA, USA) are a few examples of commercial products. The well-known aerogel-based thermal insulation product Pyrogel^®^ (Aspen Aerogels, Thermablok^®^, Hopkinton, MA, USA) has been utilized in oil and gas pipelines, energy-efficient buildings, and airplanes. According to reports, Pyrogel^®^ (Northborough, MA, USA) has a thermal conductivity as low as 0.015 W/m·K, which is much lower than that of conventional insulation materials [150]. An aerogel-based insulation product called Thermablok^®^ is mostly utilized in construction to lessen thermal bridging. Because it uses flexible sheets with aerogel grains, it offers superior insulation without taking up a lot of room [151]. Aerogel Technologies manufactures industrial blanket insulation using aerogel composites. Because of their flexibility, the materials offer excellent insulation in both hot and cold climates [152].

In recent years, numerous patent applications have also been made to improve the functionality and production methods of fiber–aerogel composites and commercialized goods. Enhancing aerogel synthesis and adding sophisticated fibers, like carbon or ceramic fibers, to increase strength and heat resistance, are just two examples of these developments.

The current US11130895B2 patent disclosure can offer aerogel compositions that are long-lasting, manageable, and have a heat storage capacity. Aerogel compositions that contain PCM coatings, particle mixes, or PCM elements trapped inside an aerogel composition’s porous network can be made available by the current disclosure. By covering an aerogel composition with PCM materials, creating particle mixes with PCM materials, or enclosing PCM materials within the porous network of an aerogel composition, the current disclosure can offer techniques for creating aerogel compositions [153].

According to the patent US7226969B2, a material with a thermal conductivity of less than or equal to 25 mW/m K at atmospheric circumstances is created by combining aerogel particles with a polytetrafluoroethylene (PTFE) binder. With minimal or no filler particle shedding, the material is moldable or formable and can be bonded between two outer layers to create structures like tapes or composites. Composites offer the advantage of withstanding bending, stretching, or flexing without significant degradation or loss of their insulating properties [154].

## 7. Challenges and Limitations

In recent years, fiber–aerogel composites have drawn a lot of interest because of their remarkable thermal insulation qualities, particularly in applications that call for lightweight, high-performance materials. The mechanical strength and flexibility of fibers are combined with the benefits of aerogels, such as their high porosity and low heat conductivity, in these composites. The large-scale production of these materials still faces difficulties despite their potential, especially in the areas of cost, consistency, and durability. Further research is needed to optimize the mechanical and radiative properties of fiber–aerogel composites, as well as to determine their thermal stability in harsh conditions.

There are a number of obstacles to the large-scale manufacturing of fiber–aerogel composites, chief among them being cost, consistency, and durability. Approximately 48% and 44% of the total cost is attributed to the high raw material and manufacturing prices of silica aerogels, the main component. Furthermore, reinforcing is required due to the intrinsic brittleness of aerogels, which is frequently accomplished by adding fibers. Fiber-reinforced aerogels are created using methods including electrospinning and sol–gel techniques, but huge quantities still present a substantial challenge in obtaining the required structural integrity and uniform distribution [155,156].

For fiber–aerogel composites, thermal stability is crucial, particularly in harsh environments. Although pure silica aerogels have poor thermal conductivity, increased radiative heat transfer causes them to perform worse at higher temperatures. To counteract this effect, opacifiers such as SiC nanoparticles can be added. For example, a study showed that 4% SiC nanowires increased thermal stability by lowering thermal conductivity at 500 °C to 0.040 W/(m·K) in silica aerogel composites. Furthermore, these composites’ structural integrity under thermal cycling is crucial; aligned fiber composites have demonstrated better resilience to thermal degradation than their randomly oriented counterparts [157,158,159].

As well as manufacturing and thermal stability challenges, optimizing properties also still remain challenging. In particular, it is crucial to strike the ideal balance between mechanical strength and radiative qualities in fiber–aerogel composites. Increasing the fiber volume percentage can improve mechanical strength, but because it creates more solid-phase conduction routes, it may also increase thermal conductivity. On the other hand, adding opacifiers may reduce radiative heat transmission while sacrificing mechanical qualities. Therefore, to maximize the composite’s overall performance, a multimodal approach involving structural design, material selection, and processing procedures is required [23].

## 8. Future Directions and Research Gaps

Fiber–aerogel composites have attracted a lot of interest because of their exceptional thermal insulation qualities and low weight, which make them appropriate for a variety of uses in fields including buildings and aircraft. But even with their encouraging performance, there are still a number of obstacles and unmet research needs to completely optimize these materials for widespread use and large-scale manufacturing. Enhancing their thermal performance, especially under harsh circumstances, requires the creation of new fiber materials and aerogels with better radiative qualities. Simultaneously, the incorporation of innovative manufacturing techniques, such as nanotechnology and 3D printing, has the potential to improve the manufacture of these composites, making them more scalable and economical. In order to comprehend the intricate relationships between radiative heat transport and microstructure, computational modeling and multiscale analysis are essential techniques. This allows for improved material design and more precise forecasts.

The development of novel fiber materials and aerogels with improved radiative qualities is the subject of ongoing study. For example, adding SiC nanowires to silica aerogels has demonstrated potential for lowering thermal conductivity at high temperatures. According to a study, 4% SiC nanowires increased thermal stability by lowering thermal conductivity at 500 °C to 0.040 W/(m·K) in silica aerogel composites. Furthermore, materials with linked networks and core–shell structures have been produced by pyrolyzing aramid nanofiber aerogels to create carbon–ceramic fiber aerogels. These materials optimize surface impedance matching and facilitate microwave absorption in harsh settings [71,160].

The manufacturing of fiber–aerogel composites is being revolutionized by new technologies like 3D printing. With exact control over internal porosity and alignment, these techniques enable the creation of intricate, adaptable structures. According to a review, 3D printing makes production easier and lowers manufacturing costs, allowing for the development of aerogels with specialized qualities for certain uses. Additionally, the creation of 3D scaffolds and aerogels has been made easier by the use of electrospinning technology. These materials have demonstrated exceptional performance in a number of domains, such as thermal insulation and biological applications [161,162].

Predicting radiative heat flow in intricate composite materials requires advances in computational modeling. The radiative characteristics and heat transfer in fiber-loaded silica aerogel composites have been investigated numerically using modified anomalous diffraction theory, which has shed light on the combined conduction and radiation heat transfer mechanisms. In order to help develop materials with optimal thermal performance, finite element modeling has also been used to simulate high-temperature heat transport in aerogel–fiber composite mats [42,45,163].

Understanding the interaction between radiative heat transmission and microstructure in fiber–aerogel composites requires multiscale study. In order to assess strain distribution and damage progression, a study put forth a multiscale framework for studying composite constructions at three different scales, ranging from microscale to macroscale. The design of composites with improved thermal insulation qualities is informed by this method’s ability to provide a thorough understanding of material behavior under varied situations [131,164].

Advancing aerogel technology requires a deliberate focus on the sustainability of its constituent materials to address escalating environmental challenges. The creation of bio-based aerogels from renewable materials such as lignin, cellulose, and chitosan is one potential field. These materials have a smaller environmental impact because of their superior thermal insulating qualities and biodegradability. For example, lignin-based aerogels can be used for a variety of thermal insulation applications because they have shown thermal conductivities as low as 0.021–0.023 W/(m·K) [165].

In addition to bio-based aerogels, fiber–opacifier systems have the potential to improve the radiative heat transfer capabilities of aerogels. The thermal performance of these composites can be greatly enhanced by combining TiO_2_ opacifiers with fiber materials like glass and ceramic, which increases their effectiveness for high-temperature insulation. The potential for high-performance applications has been demonstrated by the notable low thermal conductivity and enhanced thermal stability of mullite fiber felts impregnated with Y_2_SiO_5_ aerogels and TiO_2_ [166].

Additionally, studies are coming closer to achieving fully reprocessable and recyclable aerogels. One such instance of dynamic balance techniques being used to produce materials that maintain performance while being completely recyclable, in line with the ideas of the circular economy, is the incorporation of Kevlar nanofibers into aerogels. These advancements are essential for increasing sustainability and reducing waste [167].

The cost-effectiveness, regulatory compliance, and manufacturing scaling up of these sustainable aerogels still present difficulties, though. Future studies should concentrate on enhancing production methods, looking into substitute feedstocks, and carrying out life cycle analyses to confirm these materials’ environmental sustainability [168]. In conclusion, an intriguing route to more environmentally friendly thermal insulation solutions is the incorporation of recyclable systems and bio-based materials into aerogel technology. Overcoming the obstacles and realizing the full potential of these materials for high-performance insulation will require sustained innovation and industry cooperation.

## 9. Conclusions

This review examines the radiative heat transfer in fiber–aerogel composites. These composites are important for thermal insulation. Aerogels have a unique mesoporous structure, giving them excellent thermal properties. For instance, silica and organic aerogels have thermal conductivities at room temperature that are much lower than still air.

There are key differences in radiative heat transfer between optically thick and thin aerogels. In thick aerogels, radiative heat transfer is a material property. In thin, semitransparent aerogels, it acts as a nonlocal phenomenon. Thus, the apparent thermal conductivity measured in experiments depends on the setup, not just the material.

Combining aerogels with fibrous materials has enhanced the mechanical properties of composites. This has not significantly reduced their thermal insulation capabilities. To boost high-temperature insulation performance, new methods have been used to reduce radiative heat transfer. This includes adding infrared opacifiers like SiC, TiO_2_, and carbon. For example, ultralight ceramic fiber aerogels with SiC opacifiers have shown great potential. They can achieve thermal conductivities as low as 0.0663 W·m^−1^·K^−1^ at 1000 °C [23].

Optimizing the synthesis and manufacturing of fiber–aerogel composites remains challenging despite recent advances. We need standardized methods to analyze thermal performance accurately. This is important because of the many different experimental setups and measuring techniques. Additionally, we must carefully balance mechanical strength, flexibility, and heat conductivity when creating new aerogels and adding advanced opacifiers.

Going forward, a number of crucial research topics ought to be given top priority in order to enhance the creation and use of fiber–aerogel composites. The industrial usage of these materials depends on the development of scalable manufacturing procedures. While roll-to-roll additive manufacturing and ambient pressure drying are promising techniques, they require additional development to lower production costs and enhance material uniformity at industrial scales. The commercial viability of fiber–aerogel composites could be greatly increased by these methods, especially for use in construction and energy-efficient building materials [139]. Furthermore, in order to take into consideration the intricate relationships between light and the porous structure of the aerogels, the radiative heat transfer models that govern these materials must be enhanced. Improved models will make it easier to forecast how fiber–aerogel composites will function thermally under various circumstances, especially for semitransparent aerogels where heat transmission is dependent on the radiation wavelengths involved as well as the material [169]. Additionally, by improving the scattering and absorption of infrared radiation, advanced opacifiers and nanomaterials like graphene or metallic nanoparticles can greatly improve radiative heat transfer properties while lowering thermal conductivity without sacrificing mechanical strength. To optimize these compounds’ efficacy, future studies should concentrate on adjusting their concentration and dispersion [140]. The creation of bio-based aerogels and environmentally friendly opacifiers has to be given top priority since sustainability and environmental effects are becoming more and more significant. To ensure their wider use, particularly in green building materials and sustainable energy systems, research into the lifetime analysis of fiber–aerogel composites and the environmental impact of production methods will be essential [170]. Finally, whereas short-term experiments have demonstrated the promise of fiber–aerogel composites, long-term performance evaluations are necessary to evaluate their stability and longevity in practical settings. This includes being aware of how resistant they are to external elements like moisture, UV rays, and temperature fluctuations. Long-term testing will assist in guaranteeing these composites’ dependability in real-world uses such electronics heat management systems, energy-efficient buildings, and aircraft [132].

More complex computational models should be developed in future studies to forecast the radiative heat transfer characteristics of fiber–aerogel composites. Furthermore, the environmental sustainability of these composites may be enhanced by investigating affordable and sustainable components like bio-based fibers and opacifiers. Fiber–aerogel composites’ performance and scalability will be greatly improved by resolving these issues. The next generation of thermal insulation will also be developed based on improvements in synthesis techniques and a better comprehension of the physics of the materials. By addressing the changing needs of sectors like aerospace and construction, these developments will open up new markets for high-performance thermal insulation.

## Figures and Tables

**Table 1:** Classification of materials based on their structure, important features, and temperature range of use.

Material Type	Structure	Typical Temperature Range	Key Properties	References
Fibrous-Aerogel Composites	A network of fibers implanted in an aerogel matrix	Up to 1200–1500 °C	Lightweight, flexible, extremely low heat conductivity, and strong mechanical properties at high temperatures	[17,79]
Polyimide Aerogels	Network of polyimide polymers	Up to 1000 °C	Excellent mechanical qualities, low density, and high thermal stability	[80,81,82]
Zirconia Aerogels	Zirconia-based aerogel structure	Up to 1300 °C	Low heat conductivity and outstanding thermal resistance	[82]
Silica Aerogels	Silica-based aerogel network	Up to 1500 °C	High porosity, extremely low heat conductivity, and brittle nature	
Alumina Aerogels	Alumina-based aerogel structure	Up to 1800 °C	Low heat conductivity, high thermal stability, and adaptability to harsh environments	
Carbon Aerogels	Carbon-based aerogel network	Up to 2500 °C	Superior mechanical strength, low heat conductivity, and exceptional heat resistance	[82]
Mineral Wool	Fibrous mineral-based structure	Up to 1000–1650 °C	Cost-effective, fire-resistant, and with good thermal insulation	[83]
Fiberglass	Glass fiber network	Up to 230–260 °C	Cost-effective, lightweight, and well-insulated from heat	[84,85]
Polyurethane Foam	Polymer foam structure	Up to 120 °C	Flexibility, low heat conductivity, and light weight	[86]
Panels for Vacuum Insulation	Core material in vacuum-sealed envelope	Up to 120 °C	High insulation performance, extremely low heat conductivity, and fragility	[87,88]

**Table 2:** Comparing the Properties of Radiative Heat Transfer.

Material Type	Thermal Conductivity (W/m·K)	Radiative Heat Transfer Characteristics	Reference
Fiber–Aerogel Composites	~0.02–0.03	Infrared opacifiers and high porosity reduce radiative heat transmission, making them useful in hot conditions.	[47]
Mineral Wool	~0.035–0.045	Performance may suffer at high temperatures due to moderate radiative heat transfer.	[121]
Fiberglass	~0.035–0.045	Like mineral wool, it is frequently used for insulation in buildings.	[122]
Polyurethane Foam	~0.022–0.030	At high temperatures, radiative heat transfer can be substantial despite lower thermal conductivity.	[123]
Vacuum Insulation Panels (VIPs)	~0.004–0.007	Very low thermal conductivity; great thermal insulation, yet fragile and expensive.	[124]

**Table 3:** Summary of Key Findings, Methods, Challenges, and Future Research Directions in Fiber–Aerogel Composites for Radiative Heat Transfer.

Category	Key Findings	Methods and Techniques	Challenges	Future Research Directions	References
Radiative Heat Transfer Mechanisms	- Radiative heat transfer varies between optically thick and thin aerogels. - Diffusive radiative heat transfer is a property in optically thick aerogels, but nonlocal in thin, semitransparent ones.	- Fourier transform infrared (FTIR) spectroscopy for measuring absorption. - Numerical modeling for predicting radiative heat transfer.	- Variability in experimental setups leads to challenges in accurate measurement of thermal conductivity. - Complex behavior of radiative transfer in nonlocal, thin aerogels.	- Develop standardized measurement techniques for low thermal conductivity values. - Advanced computational models to predict radiative heat transfer behavior.	[32,33,125,126]
Fiber–Aerogel Composite Materials	- Fibrous materials enhance mechanical properties without significantly compromising thermal insulation performance. - Integration of infrared opacifiers (SiC, TiO_2_, carbon) reduces radiative heat transfer.	- Composite preparation techniques involve incorporating fibers like glass, carbon, and natural fibers into aerogels. - Infrared opacifiers enhance thermal insulation.	- Need for optimization of the aerogel–fiber ratio to balance thermal and mechanical properties. - Difficulty in processing high-temperature-resistant composites.	- Synthesis of new aerogel types with improved properties. - Explore new opacifiers for improved radiative heat transfer suppression.	[108,127,128]
Experimental Methods	- FTIR spectroscopy effectively measures absorption coefficients. - Numerical simulations can predict radiative heat transfer in complex composites.	- Use of FTIR to measure absorption properties. - Numerical simulations (e.g., Monte Carlo methods, Rayleigh scattering models) for radiative heat transfer.	- Experimental setups and results may vary, leading to inconsistencies. - High uncertainty in predicting behavior of composites under extreme conditions.	- Refining numerical models to handle various composite structures and conditions. - Enhance experimental methods for accurate heat transfer data at extreme temperatures.	[6,45,129,130,131]
High-Temperature Applications	- Fiber–aerogel composites are viable for high-temperature insulation (e.g., aerospace, energy-efficient buildings). - Aerogels can provide low thermal conductivity even in extreme environments.	- Experimentation under high-temperature conditions for insulation performance. - Thermo-gravimetric analysis (TGA) to assess stability.	- Maintaining low thermal conductivity at high temperatures. - Ensuring long-term stability of aerogels in demanding applications.	- Focus on aerospace and thermal management systems. - Exploration of new opacifiers and fiber materials for high-temperature applications.	[30,104,132,133]
Future Directions and Innovations	- Continued development of sustainable materials for aerogel composites. - New techniques in 3D printing and nanotechnology are expected to improve manufacturing	- 3D printing of aerogel composites. - Integration of nanotechnology to enhance aerogel properties.	- High production costs and complex processing techniques. - Development of scalable, reproducible aerogels for mass production.	- Innovation in synthesis techniques and advanced materials. - Improve manufacturing processes for large-scale production and cost reduction.	[134,135,136,137]

## Data Availability

No new data were created or analyzed in this study. Data sharing is not applicable to this article.

## References

[1] Kistler S.S. (1931). Coherent Expanded Aerogels and Jellies. Nature.

[2] Hrubesh L.W., Pekala R.W. (1994). Thermal properties of organic and inorganic aerogels. J. Mater. Res..

[3] Lei J., Zheng S., Han Z., Niu Y., Pan D., Liu H., Liu C., Shen C. (2024). A Brief Review on the Preparation and Application of Silica Aerogel. Eng. Sci..

[4] Gurav J.L., Jung I.-K., Park H.-H., Kang E.S., Nadargi D.Y. (2010). Silica Aerogel: Synthesis and Applications. J. Nanomater..

[5] Parale V.G., Kim T., Choi H., Phadtare V.D., Dhavale R.P., Kanamori K., Park H.-H. (2024). Mechanically Strengthened Aerogels Through Multiscale, Multicompositional, and Multidimensional Approaches: A Review. Adv. Mater..

[6] Zhao J.-J., Duan Y.-Y., Wang X.-D., Wang B.-X. (2012). Radiative properties and heat transfer characteristics of fiber-loaded silica aerogel composites for thermal insulation. Int. J. Heat Mass Transf..

[7] Shklover V., Braginsky L., Mishrikey M., Hafner C. (2009). Radiative heat transport in porous materials. MRS Online Proc. Libr..

[8] Militký J., Křemenáková D., Venkataraman M., Večerník J., Martínková L., Marek J. (2021). Sandwich Structures Reflecting Thermal Radiation Produced by the Human Body. Polymers.

[9] Venkataraman M., Mishra R., Militky J., Kremenakova D., Michal P. (2019). Aerogel Based High Performance Thermal Insulation Materials. IOP Conf. Ser. Mater. Sci. Eng..

[10] Venkataraman M., Mishra R., Wiener J., Militky J., Kotresh T., Vaclavik M. (2015). Novel techniques to analyse thermal performance of aerogel-treated blankets under extreme temperatures. J. Text. Inst..

[11] Tafreshi O.A., Mosanenzadeh S.G., Karamikamkar S., Saadatnia Z., Park C.B., Naguib H.E. (2022). A review on multifunctional aerogel fibers: Processing, fabrication, functionalization, and applications. Mater. Today Chem..

[12] Ebert H.-P., Aegerter M.A., Leventis N., Koebel M.M. (2011). Thermal Properties of Aerogels. Aerogels Handbook.

[13] Militky J., Bajzík V. (2003). Surface roughness of heat protective clothing textiles. Int. J. Cloth. Sci. Technol..

[14] Sozcu S., Frajova J., Wiener J., Venkataraman M., Tomkova B., Militky J. (2025). Synthesis of *Acetobacter xylinum* Bacterial Cellulose Aerogels and Their Effect on the Selected Properties. Gels.

[15] He Y.-L., Xie T. (2015). Advances of thermal conductivity models of nanoscale silica aerogel insulation material. Appl. Therm. Eng..

[16] Villasmil W., Fischer L.J., Worlitschek J. (2019). A review and evaluation of thermal insulation materials and methods for thermal energy storage systems. Renew. Sustain. Energy Rev..

[17] Xue J., Han R., Li Y., Zhang J., Liu J., Yang Y. (2023). Advances in multiple reinforcement strategies and applications for silica aerogel. J. Mater. Sci..

[18] Wang L., Lian W., Yin B., Liu X., Tang S. (2024). Silica nanowires-reinforced silica aerogels with outstanding thermal insulation, thermal stability and mechanical properties. Ceram. Int..

[19] Zhan W., Chen L., Kong Q., Li L., Chen M., Jiang J., Li W., Shi F., Xu Z. (2023). The Synthesis and Polymer-Reinforced Mechanical Properties of SiO_2_ Aerogels: A Review. Molecules.

[20] Deng Z., Wang J., Wu A., Shen J., Zhou B. (1998). High strength SiO_2_ aerogel insulation. J. Non-Cryst. Solids.

[21] Merillas B., Almeida C.M.R., Álvarez-Arenas T.E.G., Rodríguez-Pérez M.Á., Durães L. (2025). Enhanced thermal insulation performance of silica aerogel composites through infrared opacifier integration for high-temperature applications. Compos. Part C Open Access.

[22] Yu H., Tong Z., Zhang B., Chen Z., Li X., Su D., Ji H. (2021). Thermal radiation shielded, high strength, fire resistant fiber/nanorod/aerogel composites fabricated by in-situ growth of TiO_2_ nanorods for thermal insulation. Chem. Eng. J..

[23] Liu F., He C., Jiang Y., Feng J., Li L., Tang G., Feng J. (2023). Ultralight Ceramic Fiber Aerogel for High-Temperature Thermal Superinsulation. Nanomaterials.

[24] Fedyukhin A.V., Strogonov K.V., Soloveva O.V., Solovev S.A., Akhmetova I.G., Berardi U., Zaitsev M.D., Grigorev D.V. (2022). Aerogel Product Applications for High-Temperature Thermal Insulation. Energies.

[25] Sozcu S., Venkataraman M., Wiener J., Tomkova B., Militky J., Mahmood A. (2024). Incorporation of Cellulose-Based Aerogels into the Textile Structure. Materials.

[26] Lee O.-J., Lee K.-H., Yim T.J., Kim S.Y., Yoo K.-P. (2002). Determination of mesopore size of aerogels from thermal conductivity measurements. J. Non-Cryst. Solids.

[27] Wang J., Kuhn J., Lu X. (1995). Monolithic silica aerogel insulation doped with TiO_2_ powder and ceramic fibers. J. Non-Cryst. Solids.

[28] Daryabeigi K. (2003). Heat Transfer in High-Temperature Fibrous Insulation. J. Thermophys. Heat Transf..

[29] Wei G., Liu Y., Zhang X., Yu F., Du X. (2011). Thermal conductivities study on silica aerogel and its composite insulation materials. Int. J. Heat Mass Transf..

[30] Lu G., Wang X.-D., Duan Y.-Y., Li X.-W. (2011). Effects of non-ideal structures and high temperatures on the insulation properties of aerogel-based composite materials. J. Non-Cryst. Solids.

[31] Zeng S.O., Hunt A., Greif R. (1995). Geometric structure and thermal conductivity of porous medium silica aerogel. J. Heat Transf..

[32] Wei G., Liu Y., Zhang X., Du X. (2013). Radiative heat transfer study on silica aerogel and its composite insulation materials. J. Non-Cryst. Solids.

[33] Fu Z., Corker J., Papathanasiou T., Wang Y., Zhou Y., Madyan O.A., Liao F., Fan M. (2022). Critical review on the thermal conductivity modelling of silica aerogel composites. J. Build. Eng..

[34] Zhang H., Qiao Y., Zhang X., Fang S. (2010). Structural and thermal study of highly porous nanocomposite SiO_2_-based aerogels. J. Non-Cryst. Solids.

[35] Karadagli I., Schulz B., Schestakow M., Milow B., Gries T., Ratke L. (2015). Production of porous cellulose aerogel fibers by an extrusion process. J. Supercrit. Fluids.

[36] Xiao L., Grogan M.D., Leon-Saval S.G., Williams R., England R., Wadsworth W.J., Birks T.A. (2009). Tapered fibers embedded in silica aerogel. Opt. Lett..

[37] Sheng Z., Liu Z., Hou Y., Jiang H., Li Y., Li G., Zhang X. (2023). The Rising Aerogel Fibers: Status, Challenges, and Opportunities. Adv. Sci..

[38] Carvajal S.A., Daryabeigi K., Ramírez J.H. (2024). Predictive radiation heat transfer modeling in fibrous insulation at high temperature. Int. J. Therm. Sci..

[39] Carvajal S.A., Paulien L., Elniski A., Daryabeigi K., Berg M.J. (2025). Analytical models of radiative transfer in fibrous insulation under collimated irradiation. Int. J. Heat Mass Transf..

[40] Padmanabhan S.K., Haq E.U., Licciulli A. (2016). Synthesis of silica cryogel-glass fiber blanket by vacuum drying. Ceram. Int..

[41] Xue J., Han R., Ge Y., Liu L., Yang Y. (2024). Preparation, mechanical, acoustic and thermal properties of silica composite aerogel using wet-laid glass fiber felt as scaffold. Compos. Part A Appl. Sci. Manuf..

[42] He S., Li H., Zhang Y., Huang Y., Pan Y. (2025). High accuracy heat transfer model for aerogel/fiber composite mats. Int. Commun. Heat Mass Transf..

[43] Bi C., Tang G.H., Hu Z.J., Yang H.L., Li J.N. (2014). Coupling model for heat transfer between solid and gas phases in aerogel and experimental investigation. Int. J. Heat Mass Transf..

[44] Zhao J.-J., Duan Y.-Y., Wang X.-D., Wang B.-X. (2012). Effects of solid–gas coupling and pore and particle microstructures on the effective gaseous thermal conductivity in aerogels. J. Nanopart. Res..

[45] Xie T., He Y.-L. (2016). Heat transfer characteristics of silica aerogel composite materials: Structure reconstruction and numerical modeling. Int. J. Heat Mass Transf..

[46] Xu H.-B., Zhu C.-Y., Tian L., Li Z.-Y. (2025). Applicable scope of the Rosseland model in predicting the radiative thermal conductivity of silica aerogel. Int. J. Therm. Sci..

[47] Zhang H., Wang X., Li Y. (2018). Measuring radiative properties of silica aerogel composite from FTIR transmittance test using KBr as diluents. Exp. Therm. Fluid Sci..

[48] Wei G., Liu Y., Zhang X., Du X. Thermal Radiation in Silica Aerogel and its Composite Insulation Materials. Proceedings of the ASME 2011 International Mechanical Engineering Congress and Exposition. Volume 10: Heat and Mass Transport Processes, Parts A and B.

[49] Huang R., Jiang Y., Feng J., Li L., Hu Y., Wang X., Feng J. (2024). Robust and exceptional thermal insulating alumina-silica aerogel composites reinforced by ultra IR-opacified ZrO_2_ nanofibers. Chem. Eng. J..

[50] Yang Z., Su G., Sun F. (2013). Theoretical Modeling of the Radiative Properties and Effective Thermal Conductivity of the Opacified Silica Aerogel. CMC.

[51] Pang H.-Q., Fan T.-H., Zhu C.-Y., Liu T.-Y., Gao Y.-F. (2022). Representation of the Characteristic Temperature of Correlative Thermal Conductivity of Opacifier-Fiber Doped Silica Aerogel by Steady-State Method at Large Temperature Differences. Int. J. Thermophys..

[52] He S., Zhang X., Wu X., Li P., Xu L. (2024). Theoretical study of heat transfer model of silica aerogel based on the porous structure of secondary particles. Appl. Therm. Eng..

[53] Xiong X., Venkataraman M., Jašíková D., Yang T., Mishra R., Militký J., Petrů M. (2021). An experimental evaluation of convective heat transfer in multi-layered fibrous materials composed by different middle layer structures. J. Ind. Text..

[54] Krzemińska S., Cieślak M., Kamińska I., Nejman A. (2020). Application of Silica Aerogel in Composites Protecting Against Thermal Radiation. Autex Res. J..

[55] Goryunova K.I., Gahramanli Y.N. (2024). Insulating materials based on silica aerogel composites: Synthesis, properties and application. RSC Adv..

[56] Xiaoman X., Venkataraman M., Jašíková D., Yang T., Rajesh M., Militky J., Petru M. (2021). Thermal Behavior of Aerogel-Embedded Nonwovens in Cross Airflow. Autex Res. J..

[57] Burgos M.I., Velasco M.I., Acosta R.H., Perillo M.A. (2016). Environmental Topology and Water Availability Modulates the Catalytic Activity of β-Galactosidase Entrapped in a Nanosporous Silicate Matrix. Sci. Rep..

[58] Venkataraman M., Militký J., Mishra R., Jandová S. (2018). Unconventional measurement methods and simulation of aerogel assisted thermoregulation. J. Mech. Eng. (JMechE).

[59] Zhang H., Li Y., Tao W. (2017). Effect of radiative heat transfer on determining thermal conductivity of semi-transparent materials using transient plane source method. Appl. Therm. Eng..

[60] Al-Homoud M.S. (2005). Performance characteristics and practical applications of common building thermal insulation materials. Build. Environ..

[61] Smith D.S., Alzina A., Bourret J., Nait-Ali B., Pennec F., Tessier-Doyen N., Otsu K., Matsubara H., Elser P., Gonzenbach U.T. (2013). Thermal conductivity of porous materials. J. Mater. Res..

[62] Howell J.R., Menguc M.P., Siegel R. (2015). Thermal Radiation Heat Transfer.

[63] Lee S.W., Lim C.H., Salleh E.@.I.B. (2016). Reflective thermal insulation systems in building: A review on radiant barrier and reflective insulation. Renew. Sustain. Energy Rev..

[64] Malakooti S., Vivod S.L., Pereira M., Ruggeri C.R., Revilock D.M., Scheiman D.A., Guo H., Salem J.A., Benafan O., Johnston J.C. (2023). Fabric reinforced polyimide aerogel matrix composites with low thermal conductivity, high flexural strength, and high sound absorption coefficient. Compos. Part B Eng..

[65] Zhan C., Lu Q., Jiang H., Lu H., Liu Y. (2025). Facile preparation of lightweight high-elastic celluous/SiO_2_ composite aerogel with outstanding thermal insulation performance. J. Porous Mater..

[66] Cai H., Jiang Y., Feng J., Zhang S., Peng F., Xiao Y., Li L., Feng J. (2020). Preparation of silica aerogels with high temperature resistance and low thermal conductivity by monodispersed silica sol. Mater. Des..

[67] Lee S.C. (1989). Effect of fiber orientation on thermal radiation in fibrous media. Int. J. Heat Mass Transf..

[68] Venkataraman M., Mishra R., Kotresh T.M., Sakoi T., Militky J. (2016). Effect of compressibility on heat transport phenomena in aerogel-treated nonwoven fabrics. J. Text. Inst..

[69] Ma Y., Tang G.H., Hu Y. (2024). Modelling of hollow-fiber doping in silica aerogel composites for radiative and conductive insulation under high temperatures. Appl. Therm. Eng..

[70] Dai Y., He Y., Yu D., Dai J., Wang Y., Bai F. (2024). Study on the effect of semi-transparency on thermal insulation performance of silica aerogel composites. Case Stud. Therm. Eng..

[71] Feng T., Nie Z., Guo X., Yang X., Su K., Qi S., Cheng B. (2025). Aramid nanofibrous aerogels and their phase-change composites for highly efficient thermal management. Compos. Commun..

[72] Venkataraman M., Mishra R., Subramaniam V., Gnanamani A., Kotresh T.M., Militky J. (2016). Dynamic heat flux measurement for advanced insulation materials. Fibers Polym..

[73] 5.3.4. Rosseland Radiation Model Theory. https://ansyshelp.ansys.com/public//Views/Secured/corp/v242/en/flu_th/flu_th_sec_mod_ross.html?utm_source=chatgpt.com.

[74] Dombrovsky L.A. (2011). Diffusion Approximation in Multidimensional Radiative Transfer Problems.

[75] Zhao S., Dong J., Monte C., Sun X., Zhang W. (2020). New phase function development and complete spectral radiative properties measurements of aerogel infused fibrous blanket based on simulated annealing algorithm. Int. J. Therm. Sci..

[76] Retailleau F., Allheily V., Merlat L., Henry J.-F., Randrianalisoa J.H. (2020). Experimental characterization of radiative transfer in semi-transparent composite materials with rough boundaries. J. Quant. Spectrosc. Radiat. Transf..

[77] Huang B., Li J., Gong L., Dai P., Zhu C. (2024). The Influence of Reinforced Fibers and Opacifiers on the Effective Thermal Conductivity of Silica Aerogels. Gels.

[78] Daoût C., Rozenbaum O., De Sousa Meneses D., Rochais D. (2024). Identification of the spectral complex refractive indices of micrometric phases within a semi-transparent medium up to elevated temperatures. Int. J. Heat Mass Transf..

[79] Markevicius G., Ladj R., Niemeyer P., Budtova T., Rigacci A. (2017). Ambient-dried thermal superinsulating monolithic silica-based aerogels with short cellulosic fibers. J. Mater. Sci..

[80] Fan W., Zhang X., Zhang Y., Zhang Y., Liu T. (2019). Lightweight, strong, and super-thermal insulating polyimide composite aerogels under high temperature. Compos. Sci. Technol..

[81] Hou X., Mao Y., Zhang R., Fang D. (2021). Super-flexible polyimide nanofiber cross-linked polyimide aerogel membranes for high efficient flexible thermal protection. Chem. Eng. J..

[82] Wang C., Bai L., Xu H., Qin S., Li Y., Zhang G. (2024). A Review of High-Temperature Aerogels: Composition, Mechanisms, and Properties. Gels.

[83] Krasnovskih M.P., Maksimovich N.G., Vaisman Y.I., Ketov A.A. (2014). Thermal stability of mineral-wool heat-insulating materials. Russ. J. Appl. Chem..

[84] Zhou T., Cheng X., Pan Y., Li C., Gong L., Zhang H. (2018). Mechanical performance and thermal stability of glass fiber reinforced silica aerogel composites based on co-precursor method by freeze drying. Appl. Surf. Sci..

[85] Xia C., Hao M., Liu W., Zhang X., Miao Y., Ma C., Gao F. (2023). Synthesis of Al_2_O_3_-SiO_2_ aerogel from water glass with high thermal stability and low thermal conductivity. J. Sol-Gel Sci. Technol..

[86] Wang J., Zhang C., Deng Y., Zhang P. (2022). A Review of Research on the Effect of Temperature on the Properties of Polyurethane Foams. Polymers.

[87] Lorenzati A., Fantucci S., Capozzoli A., Perino M. (2017). The Effect of Temperature on Thermal Performance of Fumed Silica Based Vacuum Insulation Panels for Buildings. Energy Procedia.

[88] Kaushik D., Singh H., Tassou S.A. (2024). Vacuum insulation panels for high-temperature applications—Design principles, challenges and pathways. Therm. Sci. Eng. Prog..

[89] Salosina M.O., Alifanov O.M., Nenarokomov A.V. (2024). Designing Thermal Shield with Choice of Structure Parameters of Composite Based on Carbon Aerogel. J. Engin. Thermophys..

[90] Zhao J.-J., Duan Y.-Y., Wang X.-D., Wang B.-X. (2012). An analytical model for combined radiative and conductive heat transfer in fiber-loaded silica aerogels. J. Non-Cryst. Solids.

[91] Aerogel: From the Nanomaze to Global Thermal Management—How the Microporous Structure Reinvents the Laws of Heat Transfer. https://insulatewool.com/news/aerogel-from-the-nanomaze-to-global-thermal-management-how-the-microporous-structure-reinvents-the-laws-of-heat-transfer?utm_source=chatgpt.com.

[92] Arambakam R., Tafreshi H.V., Pourdeyhimi B. (2013). Dual-scale 3-D approach for modeling radiative heat transfer in fibrous insulations. Int. J. Heat Mass Transf..

[93] Venkataraman M., Mishra R., Militky J., Behera B.K. (2017). Modelling and simulation of heat transfer by convection in aerogel treated nonwovens. J. Text. Inst..

[94] Dent R.W., Skelton J., Donovan J.G. (1990). Radiant Heat Transfer in Extremely Low Density Fibrous Assemblies. Insulation Materials, Testing and Applications.

[95] Li X. (2007). Radiative Heat Transfer Through Fibrous Materials. Doctoral Thesis.

[96] Yang F., Xie W., Meng S. (2024). Effect of porous microstructure and fiber arrangement of thermal protection composites on effective thermal conductivity. Mech. Mater..

[97] Song W.F., Yu W.D. (2011). Study on radiative heat transfer property of fiber assemblies using FTIR. J. Therm. Anal. Calorim..

[98] Yuan H., Zhang H., Huang K., Cheng Y., Wang K., Cheng S., Li W., Jiang J., Li J., Tu C. (2022). Dual-Emitter Graphene Glass Fiber Fabric for Radiant Heating. ACS Nano.

[99] Wang F., Cheng L., Zhang Q., Zhang L. (2014). Effects of heat treatment and coatings on the infrared emissivity properties of carbon fibers. J. Mater. Res..

[100] Yang L., He X., He F. (2008). ITO coated quartz fibers for heat radiative applications. Mater. Lett..

[101] Veiseh S., Hakkaki-Fard A. (2009). Numerical Modeling of Combined Radiation and Conduction Heat Transfer in Mineral Wool Insulations. Heat Transf. Eng..

[102] Kang D., Jia S., Zhao C., Ni Y., Qi J., Kang Z., Sui Y., Wei F., Xiao B., Meng Q. (2024). High-temperature resistance performance of silica aerogel composites through fiber reinforcement. Ceram. Int..

[103] Saleh M.H., Dhaef A.H. (2015). Heat Transfer in Inclined Enclosure of Silica Aerogel/Glass Fiber Composite Material. Int. J. Comput. Appl..

[104] Liu H., Liu J., Tian Y., Wu X., Li Z. (2023). Investigation of high temperature thermal insulation performance of fiber-reinforced silica aerogel composites. Int. J. Therm. Sci..

[105] Zhang H., Fang W.-Z., Wang X., Li Y.-M., Tao W.-Q. (2017). Thermal conductivity of fiber and opacifier loaded silica aerogel composite. Int. J. Heat Mass Transf..

[106] Wu Q., Yang L., Chen Z., Yang M., Liu T., Li M., Mukhopadhyaya P. (2023). SiO_2_ aerogel multiscale reinforced by glass fibers and SiC nanowhiskers for thermal insulation. J. Porous Mater..

[107] Cherunova I., Kornev N., Jia G., Richter K., Plentz J. (2023). Development of Infrared Reflective Textiles and Simulation of Their Effect in Cold-Protection Garments. Appl. Sci..

[108] Lee K.H., Arshad Z., Dahshan A., Alshareef M., Alsulami Q.A., Bibi A., Lee E.-J., Nawaz M., Zubair U., Javid A. (2023). Porous Aerogel Structures as Promising Materials for Photocatalysis, Thermal Insulation Textiles, and Technical Applications: A Review. Catalysts.

[109] Yang J., Wu H., He S., Wang M. (2015). Prediction of Thermal Conductivity of Fiber/Aerogel Composites for Optimal Thermal Insulation. JPM.

[110] Gurav J.L., Rao A.V., Rao A.P., Nadargi D.Y., Bhagat S.D. (2009). Physical properties of sodium silicate based silica aerogels prepared by single step sol–gel process dried at ambient pressure. J. Alloys Compd..

[111] Li Z., Wang Y., Wu X., Liu Q., Li M., Shi L., Cheng X. (2023). Surface chemistry, skeleton structure and thermal safety of methylsilyl modified silica aerogels by heat treatment in an argon atmosphere. J. Non-Cryst. Solids.

[112] Li Z., Shen K., Hu M., Shulga Y.M., Chen Z., Liu Q., Li M., Wu X. (2023). Heat-Treated Aramid Pulp/Silica Aerogel Composites with Improved Thermal Stability and Thermal Insulation. Gels.

[113] Cuce E., Cuce P.M., Wood C.J., Riffat S.B. (2014). Toward aerogel based thermal superinsulation in buildings: A comprehensive review. Renew. Sustain. Energy Rev..

[114] Sozcu S., Frajova J., Wiener J., Venkataraman M., Tomkova B., Militky J. (2024). Effect of Drying Methods on the Thermal and Mechanical Behavior of Bacterial Cellulose Aerogel. Gels.

[115] Wang Z., Yang H., Li Y., Zheng X. (2020). Robust Silk Fibroin/Graphene Oxide Aerogel Fiber for Radiative Heating Textiles. ACS Appl. Mater. Interfaces.

[116] Sun X., Tang H., Yuan G. (2008). Anomalous diffraction approximation method for retrieval of spherical and spheroidal particle size distributions in total light scattering. J. Quant. Spectrosc. Radiat. Transf..

[117] Zhang B.-M., Zhao S.-Y., He X.-D. (2008). Experimental and theoretical studies on high-temperature thermal properties of fibrous insulation. J. Quant. Spectrosc. Radiat. Transf..

[118] Lind A.C., Greenberg J.M. (1966). Electromagnetic Scattering by Obliquely Oriented Cylinders. J. Appl. Phys..

[119] Han M., Hao M., Li Z., Jian S., Ma C., Miao Y. (2025). Ultra-light, flame-retardant nano-TiO_2_ coated silica-zirconia ceramic fiber aerogel for thermal insulation. J. Porous Mater..

[120] Hoseini A., McCague C., Andisheh-Tadbir M., Bahrami M. (2016). Aerogel blankets: From mathematical modeling to material characterization and experimental analysis. Int. J. Heat Mass Transf..

[121] Ablaoui E.M., Malendowski M., Szymkuc W., Pozorski Z. (2023). Determination of Thermal Properties of Mineral Wool Required for the Safety Analysis of Sandwich Panels Subjected to Fire Loads. Materials.

[122] Choudhary M.K., Eastes W. (2024). Effective thermal conductivity of fiberglass insulation. Int. J. Appl. Glass Sci..

[123] Zhang H., Fang W.-Z., Li Y.-M., Tao W.-Q. (2017). Experimental study of the thermal conductivity of polyurethane foams. Appl. Therm. Eng..

[124] Li X., Peng C., Liu L. (2020). Experimental study of the thermal performance of a building wall with vacuum insulation panels and extruded polystyrene foams. Appl. Therm. Eng..

[125] Meliță L., Croitoru C. (2019). Aerogel, a high performance material for thermal insulation—A brief overview of the building applications. E3S Web Conf..

[126] Jelle B.P., Baetens R., Gustavsen A. (2015). Aerogel Insulation for Building Applications. The Sol-Gel Handbook.

[127] Nannan Z., Chinese Academy of Sciences Advanced Aerogel Composite Developed for Extreme Thermal Environments. https://phys.org/news/2025-04-advanced-aerogel-composite-extreme-thermal.html.

[128] Shang L., Lyu Y., Han W. (2019). Microstructure and Thermal Insulation Property of Silica Composite Aerogel. Materials.

[129] Liu R., Dong X., Xie S., Jia T., Xue Y., Liu J., Jing W., Guo A. (2019). Ultralight, thermal insulating, and high-temperature-resistant mullite-based nanofibrous aerogels. Chem. Eng. J..

[130] Nocentini K., Ibrahim M., Biwole P.H., Achard P. (2022). Multi-scale thermal, energetic and economic analysis of composite insulating materials made of silica aerogel in a fibrous inorganic mat. Energy Build..

[131] Lakatos Á., Trník A. (2020). Thermal Diffusion in Fibrous Aerogel Blankets. Energies.

[132] Kovács Z., Csík A., Lakatos Á. (2023). Thermal stability investigations of different aerogel insulation materials at elevated temperature. Therm. Sci. Eng. Prog..

[133] Yue J., Qin M., Yu H., He Q., Feng W. (2025). Superelastic Graphene-Based Composite Aerogel for Thermal and Electromagnetic Protection in Extreme Temperature Environments. Adv. Funct. Mater..

[134] Yu D., Xue T., Ma Z., Hu Z., Long L., Miao Y.-E., Fan W., Liu T. (2024). 3D Printed Polyimide/Silica Composite Aerogels for Customizable Thermal Insulation from −50 °C to 1300 °C. Chin. J. Polym. Sci..

[135] Zhao G., Shi L., Yang G., Zhuang X., Cheng B. (2023). 3D fibrous aerogels from 1D polymer nanofibers for energy and environmental applications. J. Mater. Chem. A.

[136] Wang Z., Huang C., Han X., Li S., Wang Z., Huang J., Liu H., Chen Z. (2022). Fabrication of aerogel scaffolds with adjustable macro/micro-pore structure through 3D printing and sacrificial template method for tissue engineering. Mater. Des..

[137] Liu C., Wang S., Wang N., Yu J., Liu Y.-T., Ding B. (2022). From 1D Nanofibers to 3D Nanofibrous Aerogels: A Marvellous Evolution of Electrospun SiO_2_ Nanofibers for Emerging Applications. Nano-Micro Lett..

[138] Si Q.L., Tang G.H., Yang M.Y., Yang R., Hu Y., Du M., Zhang H. (2024). Ambient-dried hydrophobic silica aerogels for both enhanced transparency and thermal insulation. Ceram. Int..

[139] Ren S. (2023). Scalable and Cost-Effective Roll-to-Roll Additive Manufacturing of Highly Durable and Thermal Insulating Silica-Carbon Aerogel.

[140] Zhao X., Yang F., Wang Z., Ma P., Dong W., Hou H., Fan W., Liu T. (2020). Mechanically strong and thermally insulating polyimide aerogels by homogeneity reinforcement of electrospun nanofibers. Compos. Part B Eng..

[141] Yang M., Lixia Y., Chen Z., Qiong W., Wang Y., Liu T., Li M. (2023). Flexible *Electrospun strawberry*-like structure SiO_2_ aerogel nanofibers for thermal insulation. Ceram. Int..

[142] He Y., Wu S., Yuen A.C.Y., Huang F., Boyer C., Wang C.H., Zhang J. (2022). Scalable Manufacturing Process and Multifunctional Performance of Cotton Fibre-Reinforced Poly(Lactic Acid) (PLA) Bio-Composites Coated by Graphene Oxide. Polymers.

[143] Lu L., Wang H., Yun S., Hu J., Wang M. (2024). A state-of-the-art review of novel aerogel insulation materials for building exterior walls. Energy Sources Part A Recovery Util. Environ. Eff..

[144] Illera D., Mesa J., Gomez H., Maury H. (2018). Cellulose Aerogels for Thermal Insulation in Buildings: Trends and Challenges. Coatings.

[145] Koh C.H., Schollbach K., Gauvin F., Brouwers H.J.H. (2022). Aerogel composite for cavity wall rehabilitation in the Netherlands: Material characterization and thermal comfort assessment. Build. Environ..

[146] Yang W., Wang Y., Liu J. (2022). Optimization of the thermal conductivity test for building insulation materials under multifactor impact. Constr. Build. Mater..

[147] Sambucci M., Savoni F., Valente M. (2023). Aerogel Technology for Thermal Insulation of Cryogenic Tanks—Numerical Analysis for Comparison with Traditional Insulating Materials. Gels.

[148] Park M. (2025). Recent Advances in Wearable Thermal Devices for Virtual and Augmented Reality. Micromachines.

[149] Chen L., Yu X., Gao M., Xu C., Zhang J., Zhang X., Zhu M., Cheng Y. (2024). Renewable biomass-based aerogels: From structural design to functional regulation. Chem. Soc. Rev..

[150] Pyrogel X.T.E. Aspen Aerogels. https://www.aerogel.com/product/pyrogel-xte/.

[151] High Performance Thermal Insulation—Thermablok Aerogel, Thermablok–Intelligent Insulation—High Performance Thermal Insulation. https://www.thermablok.co.uk/.

[152] Aerogel Technologies, LLC|Classic Aerogel Products. https://www.aerogeltechnologies.com/classic-aerogels/classic-aerogel-products/.

[153] Trifu R., Begag R., Gould G., White S. (2021). Aerogel Composites Having Thermal Storage Capacity. U.S. Patent.

[154] Ristic-Lehmann C., Farnworth B., Dutta A. (2007). Aerogel/PTFE Composite Insulating Material. U.S. Patent.

[155] Liao Y., Wu H., Ding Y., Yin S., Wang M., Cao A. (2012). Engineering thermal and mechanical properties of flexible fiber-reinforced aerogel composites. J. Sol-Gel Sci. Technol..

[156] Song Z., Lei Y., Ran W., Yuan M., Shang S., Cui S. (2024). Structural properties and barrier performance of low-cost aerogel composites for building insulation. J. Build. Eng..

[157] Wang H., Huang Y., Liu S., Gao Y., Cheng X., Meng C. (2024). A review of silica fiber-based aerogels: Composition, construction methods, mechanical enhancement strategies and applications. Eur. Polym. J..

[158] Wu Q., Yang M., Chen Z., Lu L., Ma Z., Ding Y., Yin L., Liu T., Li M., Yang L. (2025). A layered aerogel composite with silica fibers, SiC nanowires, and silica aerogels ternary networks for thermal insulation at high-temperature. J. Mater. Sci. Technol..

[159] Zhu Z., Zhang W., Huang H., Li W., Ling H., Zhang H. (2025). A Review of High-Temperature Resistant Silica Aerogels: Structural Evolution and Thermal Stability Optimization. Gels.

[160] Huang W., Yang Y., Gu H., Yu W., Shao G. (2025). A core–shell carbon–ceramic fibrous aerogel derived from aramid-polysilsesquioxane for broadband electromagnetic wave absorption. J. Mater. Chem. C.

[161] Zhang W., Wang Y., Li J. (2024). Sustainable 3D Printing Aerogel Materials and Application: A Review. IFFTI Annu. Proc..

[162] Chen Y., Shafiq M., Liu M., Morsi Y., Mo X. (2020). Advanced fabrication for electrospun three-dimensional nanofiber aerogels and scaffolds. Bioact. Mater..

[163] RMishra, Behera B.K., Muller M., Petru M. (2021). Finite element modeling based thermodynamic simulation of aerogel embedded nonwoven thermal insulation material. Int. J. Therm. Sci..

[164] Wang M. (2021). A Multiscale Method Across Three Length Scales for Progressive Damage Analysis of Plain Woven Composites. Appl Compos. Mater..

[165] Karaaslan M.A., Kadla J.F., Ko F.K., Faruk O., Sain M. (2016). 5-Lignin-Based Aerogels. Lignin in Polymer Composites.

[166] Zhang R., Gu H., Hou X., Zhou P. (2021). High-temperature resistant Y_2_SiO_5_–TiO_2_ aerogel composite for efficient thermal insulation. J. Porous Mater..

[167] Li L., Lyu J., Cheng Q., Fu C., Zhang X. (2023). Versatile Recyclable Kevlar Nanofibrous Aerogels Enabled by Destabilizing Dynamic Balance Strategy. Adv. Fiber Mater..

[168] García-González C.A., Blanco-Vales M., Barros J., Boccia A.C., Budtova T., Durães L., Erkey C., Gallo M., Herman P., Kalmár J. (2025). Review and Perspectives on the Sustainability of Organic Aerogels. ACS Sustain. Chem. Eng..

[169] An L., Wang J., Petit D., Armstrong J.N., Li C., Hu Y., Huang Y., Shao Z., Ren S. (2020). A scalable crosslinked fiberglass-aerogel thermal insulation composite. Appl. Mater. Today.

[170] Maleki H., Durães L., Portugal A. (2014). Synthesis of lightweight polymer-reinforced silica aerogels with improved mechanical and thermal insulation properties for space applications. Microporous Mesoporous Mater..

